# A non-canonical role for desmoglein-2 in endothelial cells: implications for neoangiogenesis

**DOI:** 10.1007/s10456-016-9520-y

**Published:** 2016-06-23

**Authors:** Lisa M. Ebert, Lih Y. Tan, M. Zahied Johan, Kay Khine Myo Min, Michaelia P. Cockshell, Kate A. Parham, Kelly L. Betterman, Paceman Szeto, Samantha Boyle, Lokugan Silva, Angela Peng, YouFang Zhang, Andrew Ruszkiewicz, Andrew C. W. Zannettino, Stan Gronthos, Simon Koblar, Natasha L. Harvey, Angel F. Lopez, Mark Shackleton, Claudine S. Bonder

**Affiliations:** 1 Centre for Cancer Biology, University of South Australia and SA Pathology, PO Box 14, Rundle Mall, Adelaide, SA 5000 Australia; 2 Cancer Development and Treatment Laboratory, Peter MacCallum Cancer Centre, Melbourne, VIC Australia; 3 Sir Peter MacCallum Department of Oncology, Melbourne, VIC Australia; 4 Department of Pathology, University of Melbourne, Melbourne, VIC Australia; 5 South Australian Health and Medical Research Institute, Adelaide, SA Australia; 6 Adelaide Medical School, Faculty of Health Sciences, University of Adelaide, Adelaide, SA Australia

**Keywords:** DESMOGLEIN-2, Endothelial progenitor cells, Endothelium, Hematopoietic stem and progenitor cells, Mouse models of neoangiogenesis

## Abstract

Desmogleins (DSG) are a family of cadherin adhesion proteins that were first identified in desmosomes and provide cardiomyocytes and epithelial cells with the junctional stability to tolerate mechanical stress. However, one member of this family, DSG2, is emerging as a protein with additional biological functions on a broader range of cells. Here we reveal that DSG2 is expressed by non-desmosome-forming human endothelial progenitor cells as well as their mature counterparts [endothelial cells (ECs)] in human tissue from healthy individuals and cancer patients. Analysis of normal blood and bone marrow showed that DSG2 is also expressed by CD34^+^CD45^dim^ hematopoietic progenitor cells. An inability to detect other desmosomal components, i.e., DSG1, DSG3 and desmocollin (DSC)2/3, on these cells supports a solitary role for DSG2 outside of desmosomes. Functionally, we show that CD34^+^CD45^dim^DSG2^+^ progenitor cells are multi-potent and pro-angiogenic in vitro. Using a ‘knockout-first’ approach, we generated a *Dsg2* loss-of-function strain of mice (*Dsg2*^lo/lo^) and observed that, in response to reduced levels of *Dsg2*: (i) CD31^+^ ECs in the pancreas are hypertrophic and exhibit altered morphology, (ii) bone marrow-derived endothelial colony formation is impaired, (iii) ex vivo vascular sprouting from aortic rings is reduced, and (iv) vessel formation in vitro and in vivo is attenuated. Finally, knockdown of *DSG2* in a human bone marrow EC line reveals a reduction in an in vitro angiogenesis assay as well as relocalisation of actin and VE-cadherin away from the cell junctions, reduced cell–cell adhesion and increased invasive properties by these cells. In summary, we have identified DSG2 expression in distinct progenitor cell subpopulations and show that, independent from its classical function as a component of desmosomes, this cadherin also plays a critical role in the vasculature.

**Electronic supplementary material:**

The online version of this article (doi:10.1007/s10456-016-9520-y) contains supplementary material, which is available to authorized users.

## Introduction

Desmoglein-2 (DSG2), a type 1 transmembrane protein belonging to the cadherin family, is an adhesion molecule with well-described functions in the formation of cell–cell adhesion complexes called desmosomes. The desmosome is a specialised junction that provides intercellular links giving tensile strength to tissues that experience mechanical stress, such as in the myocardium, skin and gastrointestinal mucosa [1–3]. These complex, transversely symmetrical structures are composed of the transmembrane spanning cadherins, which interact via homotypic and/or heterotypic combinations to form the adhesive interface between cells, together with the intracellular proteins plakoglobin, desmoplakin and plakophilin which link the junction to the intermediate filament cytoskeleton. In humans, four desmoglein isoforms (DSG1-4) and three desmocollin isoforms (DSC1-3) have been identified, which are temporo-spatially expressed [4]. DSG2 is expressed in all desmosome-bearing tissues, including cardiac muscle, whereas distribution of other isoforms is confined primarily to stratified epithelial tissue [1–3]. In keeping with its role in tissue integrity, loss of DSG2 function in humans is directly linked to arrhythmogenic right ventricular cardiomyopathy (ARVC), an autosomal recessive disease underpinned by myocyte apoptosis and fibrous degeneration of the myocardium [5, 6].

In the context of tissue remodelling, the identification of endothelial progenitor cells (EPCs) has significant clinical implications for the repair processes of wound healing [7], limb ischaemia [8] and myocardial ischaemia [9, 10]. Reduced numbers of circulating EPCs are commonly observed in pathological conditions such as diabetes and cardiovascular disease [11–14]. Despite ongoing controversies regarding the nature and role of EPCs in vasculogenesis [12, 15–18], the ability of human EPCs to rescue diminished blood flow in a number of animal models [19, 20] provided a rationale for clinical trials of EPC infusion therapy in humans. Pre-clinical studies found infusion of CD34^+^ and CD133^+^ EPCs to be safe and beneficial, but the effects of EPCs in humans were less robust [12]. Nevertheless, since the discovery of EPCs [21], significant steps forward have been taken to better define and functionally characterise these cells, and in the light of the enormous therapeutic potential of modulating vasculogenesis in disease, efforts continue to better understand their origin, plasticity and role in the vasculature.

In this study, we reveal that DSG2 expression and function extend beyond myocardial cells and the epithelium to hematopoietic stem and progenitor cells (HSPCs), EPCs and some mature endothelium but not mature hematopoietic cells. Through functional studies, we demonstrate that DSG2^+^ EPCs are pro-angiogenic, and the generation of a *Dsg2* loss-of-function strain of mice has revealed that this surface-expressed cadherin regulates EC morphology and is important for vascular sprouting and colony formation ex vivo, as well as vessel formation in vivo. Moreover, we demonstrate that despite ECs being a non-desmosome-forming cell, reduction of DSG2 on these cells significantly impacts on their cell–cell adhesive capacity which is likely via reduced DSG2–DSG2 homotypic interactions. Taken together, we provide novel insights into an underappreciated role for DSG2 in hematopoietic cells and the vasculature.

## Materials and methods

### Ethics statement

The collection of primary human umbilical vein endothelial cells (HUVEC), mononuclear cells (MNC) from buffy coats or freshly collected peripheral blood, human mesenchymal stromal cells, gingival and periodontal stem cells, healthy donor peripheral blood, bone marrow, normal tissue as well as cancerous tissue was approved by the Human Research Ethics Committee of the Royal Adelaide Hospital (RAH), Adelaide, South Australia. The collection of primary human umbilical cord blood (UCB) was approved by the Human Research Ethics Committee of the Children, Youth and Women’s Health Service (CYWHS), North Adelaide, South Australia. Animal experiments were approved by the Animal Ethics Committees of SA Pathology and the Peter MacCallum Cancer Centre (protocol E526) and conformed to the guidelines established by the ‘Australian Code of Practice for the Care and Use of Animals for Scientific Purposes’.

### Isolation and culture of UCB CD133^+^ non-adherent endothelial forming cells (naEFCs)

UCB (20–130 ml) was obtained from healthy pregnant women undergoing elective caesarean section and collected into MacoPharma cord blood collection bags (MSC1201DU; MacoPharma, Mouvaux, France). CD133^+^ cells were isolated prior to naEFC cell culture using published methods [22].

### Peripheral blood MNCs, human umbilical vein endothelial cells (HUVEC), bone marrow endothelial cells (BMEC) and normal human bone marrow

Peripheral blood from healthy individuals was collected in lithium heparin coated Vacuette tubes (Greiner Bio-One, Kremsmuenster, Austria) or was provided as buffy coats from the Australian Red Cross Blood Service. For most experiments, MNCs were isolated using Lymphoprep. However, for analysis of VEGFR2^+^ EPCs, whole blood was subjected to erythrocyte lysis using PharmLyse (BD, Franklin Lakes, NJ, USA) followed by depletion of mature leucocytes using the Lineage Cell Depletion kit (Miltenyi Biotec, Bergisch Gladbach, Germany) according to the manufacturer’s instructions. Primary HUVEC were extracted from human umbilical veins by collagenase digestion and cultured in HUVE medium as previously described [23, 24] and were used for no more than two passages. Human bone marrow endothelial cells (TrHBMEC) were a kind gift from B Weksler (Cornell University Medical College, NY, USA) [25, 26] and hereafter labelled as BMEC. Normal human bone marrow samples were pre-filtered through a 70-µm nylon filter (BD Falcon) to remove debris and then subject to red blood cell lysis using PharmLyse (BD) according to the manufacturer’s instructions prior to flow cytometric staining and analysis.

### Induced pluripotent, dental pulp and mesenchymal stem cells

Bone marrow-derived human mesenchymal stem cells (Merck Millipore, NSW, Aust.) were cultured as per manufacturers’ instructions. Induced pluripotent stem (iPS) cells were generated and confirmed for pluripotency as previously described [27]. Similarly, dental pulp stem cells (DPSCs) were isolated from dental pulp tissue and enzymatically digested as per previous instructions [28].

### Flow cytometric analysis of cell surface protein expression

Staining was performed in complete RF-10 medium (RPMI + 10 % FCS) with cells (<1 × 10^6^) blocked with 1 mg/ml human IgG, incubated with unconjugated mAbs (Online resource Supp. Table 1), washed and incubated with secondary antibody. After washing, 5 μl of normal mouse serum was added and conjugated mAbs were then added to the cells. After washing, cells were fixed in 1 % formaldehyde, 20 g/l glucose, 5 mM sodium azide in PBS (‘FACS-Fix’), prior to analysis using either an Accuri C6 Cytometer (BD) or Gallios Flow Cytometer (Beckman Coulter, Brea, CA, USA). Further analysis was performed using FCS Express 4 Flow Cytometry: Research Edition (De Novo Software, CA, USA).

### DSG2^+^ cell enrichment by flow cytometric sorting and expansion

DSG2^+^ cells were isolated from UCB or fresh bone marrow (BM)-derived MNCs using a FACSAria II (BD). MNCs were co-stained with anti-DSG2, anti-CD34-PerCP-Cy5.5 and anti-CD45-FITC. Within the CD34^+^CD45^dim^ population, the DSG2^+^ and DSG2^−^ cells were identified and sorted by FACS, with the sorting gate position based on the fluorescence-minus-one (FMO) control. Immediately following isolation, a small portion of the sorted cells was re-analysed on the FACSAriaTM II (BD) to assess purity. Sorted DSG2^+^ and DSG2^−^ cells were cultured in EPC expansion CellGro media (CellGenix, Freiburg, Germany) as previously described [29].

### Detection of human DSG2 mRNA using quantitative polymerase chain reaction (qPCR)

Quantification of mRNA levels was carried out using qPCR. Primers were designed for human *DSG2* (F-5′-CCATTGCGGAAAGGGCGCCA-3′, R-5′-GGCAGAAATGATGGCACCACCTTGT-3′) using Primer Blast (NIH, MD, USA) and purchased from GeneWorks (Hindmarsh, SA, Aust.). Quantitative PCR amplification was performed using QuantiTect™ SYBR Green master mix (QIAGEN, Hilden, Germany) on a Rotor-Gene thermocycler (Corbett Research, Mortlake, NSW, Aus.) with reaction parameters: 15 min at 95 °C, then cycling of 10 s 95 °C, 20 s 55 °C and 30 s 72 °C; for 45 cycles followed by a melt phase. Resultant data were analysed using Rotor-Gene Analysis Software version 6 (Corbett Research). Relative gene expression levels were calculated using standard curves generated by serial dilutions of cDNAs normalised to the human house-keeping gene cyclophilin A (*CycA*) (F-5′GGCAAATGCTGGACCCAACACAAA-3′, R-5′CTAGGCATGGGAGGGAACAAGGAA3′).

### Hematopoietic colony formation assay

Freshly isolated DSG2^+^ naEFCs (±expansion) (~1800 DSG2^+/−^ sorted and expanded cells/well or ~500 freshly sorted cells/well) were plated in methylcellulose and cultured with growth factors as previously described [22] with hematopoietic colonies scored after 14 days.

### Generation of the Dsg2 ‘loss-of-function’ mouse strain

The pipeline for the design and construction of the *Dsg2* ‘loss-of-function’ mouse strain was based on the publication by Skarnes et al. [30]. Briefly, the CSD (Children’s Hospital Oakland Research Institute/Wellcome Trust Sanger Institute/University of California at Davis) approach incorporates a promoter-driven targeting cassette for the generation of a ‘knockout-first allele’ wherein *loxP* and *FRT* sites are placed in introns of genes of interest [30]. More specifically for this study, this ‘gene trap’ approach utilised a targeted insertion of a FRT-flanked lacZ-neomycin cassette into intron 1 of the murine *Dsg2* locus of in embryonic stem (ES) cells (i.e., clone EPD0156_5_E04; EuMMCR, Neuherberg, Germany). The *Dsg2*tma1 strain of mice were generated via ES cell microinjection into Balb/c blastocysts to generate chimeric mice which were then confirmed to be heterozygous for insertion of the *lacZ*-neomycin construct prior to being inter-bred bred for germ line transmission using C57Bl/6 N mice at the Australian Phenomics Network (APN, Melbourne, Vic. Aust.). These mice yielded fertile wildtype (WT), heterozygous and homozygous offspring at the expected Mendelian ratios (1:2:1), with litter sizes similar to WT C57BL/6N controls.

### Detection of murine Dsg2: genomic DNA and mRNA

Genomic DNA was extracted from mouse-tail tips using a REDExtract-N-Amp Tissue PCR Kit (Sigma-Aldrich, St. Louis, MO, USA) prior to genotype confirmation via PCR with reaction parameters as follows: 2 min at 94 °C, then cycling of 30 s at 94 °C, 30 s at 61 °C and 45 s at 72 °C; for 35 cycles followed by 4 °C. The following primers were used to determine WT, heterozygous and homozygous *Dsg2*tma1 genotypes; P1: 5′CTGCTGCAACTTGATGTCC3′, P2: 5′GAAAGGCGGTTCTGTAATTCC3′ and P3: 5′CAACGGGTTCTTCTGTTAGTCC3′.

Total RNA was extracted from hearts and skin harvested from C57Bl/6 wildtype (WT), *Dsg2*tma1 heterozygous and homozygous mice. Hearts were homogenised in Trizol prior to chloroform extraction. The aqueous layer containing the RNA was then isolated and 70 % ethanol added before completing the extraction method using an RNEasy Mini Kit (QIAGEN). 0.5–1 μg of total RNA was converted into first-strand cDNA using Superscript III reverse transcriptase (Life Technologies, Carlsbad, CA, USA) following the manufacturer’s instructions.

*Dsg2* gene knockdown was validated by qPCR using primers designed to span an intron/exon border between exons 14 and 15 (F-5′AACGAAGCCGTAAGGACAAG3′ R-5′GCCGCTTTCTCTGTGAAGTA3′) via Primer Blast, and purchased from GeneWorks. qPCR amplification was performed using QuantiTect™ SYBR Green master mix (QIAGEN) on a Rotor-Gene thermocycler (Corbett Research) with reaction parameters: 15 min at 95 °C, then cycling of 10 s 95 °C, 20 s 55 °C and 30 s 72 °C; for 45 cycles followed by a melt phase. Data obtained were analysed using Rotor-Gene Analysis Software version 6 (Corbett Research). Relative gene expression levels were calculated by normalising to the mouse house-keeping gene *hprt* (F-5′CCCAGCGTCGTGATTAGCG3′) (R- 5′GCA CACAGAGGGCCACAATG3′) using geNorm software [31].

### Immunohistochemistry and immunofluorescence

Mouse hearts were fixed in PBS containing 4 % formaldehyde, washed and embedded in paraffin; 4-μm-thick tissue sections were cut and stained with Mayer’s hematoxylin and eosin staining (H&E) (Dako Agilent Technologies, Glostrup, Denmark).

Tissue microarray (TMA) slides were purchased from BioChain (Newark, CA, USA). Slides were dewaxed and rehydrated prior to pressure cooker antigen retrieval in 10 mM citrate buffer (pH 6) for 20 min. After cooling to 30 °C, the sections were incubated for 60 min at room temperature with primary mAb directed against DSG2 (1:50 dilution; clone 3G132; Abcam) or an isotype-matched negative control. The polymer system ADVANCE HRP (Dako Australia Pty Ltd, Vic., Australia) employing DAB as the detection system was used for detection of primary mAb binding. Counterstaining was performed using Mayer’s hematoxylin.

Immunofluorescence microscopy was performed on paraffin-embedded human cervical sections with heat mediated antigen retrieval in 10 mM citrate buffer (pH 6.5) incubated overnight at 4 °C with a primary antibody against DSG2 (2 μg/ml, clone 6D8, Invitrogen, Carlsbad, CA, USA) or CD31 (2 μg/ml, pAb, Bethyl Laboratories, Montgomery, TX, USA). Sections were washed and secondary antibodies (goat anti-mouse Alexa fluor-594 (1:500, Abcam, Cambridge, UK) or goat anti-rabbit Alexa Fluor-488 (1:500, Abcam) applied for 1 h at RT prior to washing and mounting in ProLong Gold with DAPI (Life Technologies). Sections were analysed using an Olympus CV1000 spinning disk microscope and integrated software.

For whole mount staining of adult mouse ear skin, ears were fixed in 4 % paraformaldehyde overnight at 4 °C. Skin was then peeled away from cartilage and incubated in blocking solution (PBS containing 1 % BSA and 0.3 % Triton X-100) overnight at 4 °C. Following blocking, ear skin samples were incubated with rat anti-CD31 (BioLegend, San Diego, CA, USA), rabbit anti-LYVE-1 (Angiobio, Del Mar, CA, USA) and Cy3-conjugated anti-SMA (Sigma-Aldrich) diluted in blocking solution overnight at 4 °C and were then washed extensively in PBS-0.1 % Triton X-100 (6 × 1 h) at 4 °C. Ear skin samples were then incubated with Alexa Fluor^®^-conjugated secondary antibodies (Invitrogen) overnight at 4 °C and washed as outlined above. Samples were mounted using DAPI FLUOROMOUNT™ G (ProSciTech). Images were captured using a Zeiss LSM 700 confocal microscope (Zeiss Laboratories, Jena, Germany) and a Zeiss inverted Axio Observer Z1 fluorescence microscope.

For immunofluorescence staining of mouse pancreases, mice were humanely killed via cervical dislocation and the whole pancreas was removed. Pancreases were washed in cold phosphate buffered saline (PBS; Life Technologies) and fixed in 4 % paraformaldehyde (Chem Cruz, Santa Cruz Biotechnology Inc., Dallas, TX, USA). They were then embedded in OCT (TissueTek, Sakura Finetek, Torrance, CA, USA), frozen using dry ice and cut into 5-μm-thin sections using a cryostat (CM1520, Leica Biosystems, North Ryde, NSW, Aust.). Frozen sections were probed with primary antibodies: rat anti-mouse CD31 (Biolegend), Cy-3-conjugated anti-SMA (Sigma-Aldrich) or isotype control of rat IgG (Abcam) followed by goat anti-rat Alexa Fluor-488 or -594 (Life Technologies). DAPI Pro-long gold anti-fade mounting medium (Life Technologies) was used for nuclear staining and mounting with images captured using a Zeiss LSM710 multiphoton or Olympus IX71 (Olympus Corporation, Tokyo, Japan) fluorescence microscope.

Immunofluorescence of siRNA-KD BMEC cells was undertaken by fixing cells in either 2 % paraformaldehyde or cold 1:1 acetone:methanol (for DSG2) 72 h post-transfection. After 5 % goat serum blocking, cell-containing coverslips were labelled overnight with the following mouse monoclonal antibodies: VE-cadherin (1:50; clone F-8-Santa Cruz Biotechnology) and DSG2 (1:50; clone 6D8-Thermo Fisher). Primary antibody binding was detected using 1:500 goat anti-mouse IgG conjugated to AlexaFluor 488. To label F-actin filaments, cells were incubated with 1:1000 Phalloidin-Rhodamine (Thermo Fisher) for an hour. Coverslips were mounted on glass slides in Prolong Gold Antifade Reagent with DAPI (Thermo Fisher). Images were captured using Olympus CV1000 with a 40× objective, and analysis of VE-cadherin staining was undertaken using an ImageJ macro. Staining was split into respective channels, i.e., blue (nuclei) and green (VE-cadherin). This macro-determined the total positive areas and number of nuclei per image. Results were presented as the total fluorescence area over nuclei count. To characterise cell phenotypes of DSG2-KD cells, phalloidin staining was analysed in a randomised and blinded manner. Cells were categorised and counted as either cortical actin (circumferential) or stress fibre (mild to extensive actin network) and the percentage was determined and averaged for each siRNA and control. In addition, Image J was used to determine the localisation of VE-cadherin protein via pixel intensity as previously described [34].

### Matrigel tube formation assay: in vitro

In vitro angiogenesis assay of HUVEC and DSG2^+^ sorted cells was performed using a Matrigel matrix as previously described [29]. Briefly, cells were seeded together on a layer of Matrigel (In vitro Technologies, Noble Park, Vic., Aust.) at a cell density of 1.7 × 10^4^ HUVEC, with or without 1.0 × 10^4^ DSG2^+^ sorted cells/well, in duplicate. Similarly, the BMEC cells were seeded at 1.5 × 10^4^ cells per well in triplicate. Images covering the entire well were captured after 5–6 h using an inverted imaging microscope (EVOS XL, Life technologies), merged using Adobe Photoshop and tube-like structures manually counted using Image J software (1.47v, NIH, Bethesda, MD, USA).

### Matrigel plug assay: in vivo

Female *Dsg2*^+/+^ (wildtype, WT) and *Dsg2*^lo/lo^ mice were used between 6 and 8 weeks of age and housed in specific pathogen-free conditions in the SA Pathology Animal Care Facility with Matrigel plug assays conducted as previously described [29]. Briefly, 500 µl of Matrigel containing 2 µg/ml basic fibroblast growth factor (R&D Systems, Minneapolis, MN, USA) and 50 units/ml heparin (Sigma-Aldrich) were injected subcutaneously into both flanks of 6–8 week-old mice. Mice were humanely killed by cervical dislocation on day 7 post-injection and Matrigel plugs removed, washed in PBS, fixed in 4 % paraformaldehyde (VWR International, Radnor, PA, USA) overnight, embedded in paraffin, cut into 8 µm sections on a microtome (RM 2235 Leica, Solms, Germany) and placed onto slides (Polysine^®^, Menzel-Gläser, Thermo Scientific). The sections were stained for CD31^+^ ECs (goat anti-mouse/human CD31, Santa Cruz, Dallas, TX) with biotinylated rabbit anti-goat secondary (Abcam), followed by Vectastain elite ABC regent (Vector Labs, Burlingame, CA, USA) for DAB staining as per manufacturer’s instructions and hematoxylin counterstaining. Negative controls included isotype-matched irrelevant antibodies. Images of sections were collected using a Hamamatsu Nanozoomer slide scanner with erythrocyte-containing CD31^+^ vessels counted manually and section areas quantified using the NDPview software (Hamamatsu Photonics, Hamamatsu, Japan).

### Mouse endothelial cell colony-forming assay

Using published methods, whole BM cells were flushed from femurs and tibiae of *Dsg2*^+/+^ and *Dsg2*^lo/lo^ mice and adherent EC colony units were defined as a cell mass composed of a central cord of round cells with elongated spindle-shaped cells sprouting at the periphery [32].

### Aortic ring assay

The aortic ring assay was performed on thoracic aorta-derived rings from 12- to 14 week-old *Dsg2*^+/+^ or *Dsg2*^lo/lo^ mice as described [33] where aortic rings were observed over a period of 6 days without or with VEGF (30 ng/ml) added at day three. Aortic sprouts were stained with rat anti-CD31 (BioLegend) and goat anti-rat Alexa Fluor-594 (Life Technologies) to confirm endothelialisation, captured by a Zeiss LSM 700 confocal microscope (Zeiss Laboratories, 10× obj) and quantitated using phase-contrast microscopy via an inverted IX70 microscope 4x/0.13NA obj, an S15 F view camera and Analysis Life Sciences software (Olympus). Number of rings quantitated per group was 3–4.

### B16 melanoma mouse model

To evaluate CD31^+^ vessels in tumours, 1.4 × 10^4^ B16 mouse melanoma cells were injected into the right flanks of age-matched, syngeneic (C57Bl/6 N) *Dsg2*^+/+^ (WT) and *Dsg2*^lo/lo^ mice and resultant tumours harvested at endpoint. Sections were cut from FFPE-stored tumour tissues and labelled with anti-CD31 antibody (BD Biosciences). Secondary detection was performed using biotinylated anti-rat-Ig, streptavidin-conjugated horse-radish peroxidase and 3-amino-9-ethylcarbazole chromogen. Stained sections were scanned using an Olympus VS120 scanner and CD31^+^ blood vessels were counted in each field to obtain an average number of CD31 + blood vessels per field.

### DSG2 gene knockdown using siRNA

‘Trilencer-27’ siRNA duplexes targeting human *DSG2* were purchased from Origene Technologies (Rockville, MD, USA). Transfection was performed using Lipofectamine RNAiMax (Thermo Fisher) according to the manufacturer’s protocol, using a final concentration of 10 nM for both *DSG2*-targeting and control siRNAs. On the basis of time course experiments, BMEC cells were used in experiments 72 h after knockdown where DSG2 knockdown efficiency was routinely assessed by flow cytometry.

Cell viability was assessed by incubating the cells with annexin V-FITC (1:50 in 1x binding buffer; BD Bioscience) for 15 min at RT prior to incubation with 7-AAD (BD Biosciences) for 5 min at 4 °C. Stained cells were then resuspended in FACS-Fix and analysed by flow cytometry as described above.

### EC cell adhesion assay

BMEC (±*DSG2*-targeting or control siRNA) transfected 48 h prior were cultured at 1 × 10^6^ cells/35 mm × 10 mm dish (Corning, Sigma-Aldrich) to form a confluent monolayer. Twenty-four hours later, 0.5 × 10^6^ of 72 h-transfected BMEC (±*DSG2*-targeting or control siRNA) were labelled with 0.5 µM Calcein-AM (eBiosciences, San Diego, CA, USA) and incubated on top of the confluent monolayer for 15 min after which time the non-adherent cells were washed with Hanks Balanced Salt Solution (HBSS, Sigma-Aldrich) using a parallel plate flow chamber system (GlycoTech Corporation, Gaithersburg, MD, USA) with a flow rate of 0.156 ml/min for 2 min. Fluorescently labelled adherent cells were imaged using an inverted epifluorescence microscope Olympus IX71 and a 4×/0.10 objective. Adherent cells were quantified using ImageJ in a blinded and randomised manner.

### Inverse invasion assay

Adapted from Hennigan et al. [35], 100 µl of growth factor-reduced Matrigel diluted 1:1 in cold PBS was added into an 8.0-µm-pore-sized Transwell (Corning Incorporated) and allowed to set. Transwells were then inverted, and 3000 siRNA-transfected (at 24 h) BMEC were seeded onto the underside of the membrane. Four hours later, the unbound cells were rinsed and Transwells immersed right-way up in serum-free HUVE media; 25 ng/ml VEGF in 10 % serum was added to the upper chamber as chemoattractant, and cells allowed to migrate upward into the Matrigel for 96 h. Transwells were paraformaldehyde fixed, RNAse-treated (100 µg/ml, Affymetrix USB) and stained with 0.05 mg/ml of Propidium Iodide (Thermo Fisher), and all steps were carried out for 30 min with two PBS washes between each. Transwells were imaged at fixed intervals (6.68 μm) starting at the membrane and in a direction towards the chemoattractant using z-stack setting of Zeiss LSM 700 confocal microscope with a 10× objective. Cells from 3 fields of view per slice of distance travelled were quantified using ImageJ and then averaged.

### Statistical analysis

Results were expressed as mean ± standard error of the mean (SEM) from at least 3 experiments. Unless otherwise stated, an unpaired Student’s *t* test, 1- or 2-way ANOVA for multiple comparisons, was performed to determine statistical significance between groups with *p* values <0.05 considered significant.

## Results

### Human endothelial progenitor cells (EPCs) express desmoglein (DSG)-2

**Fig. 1:**
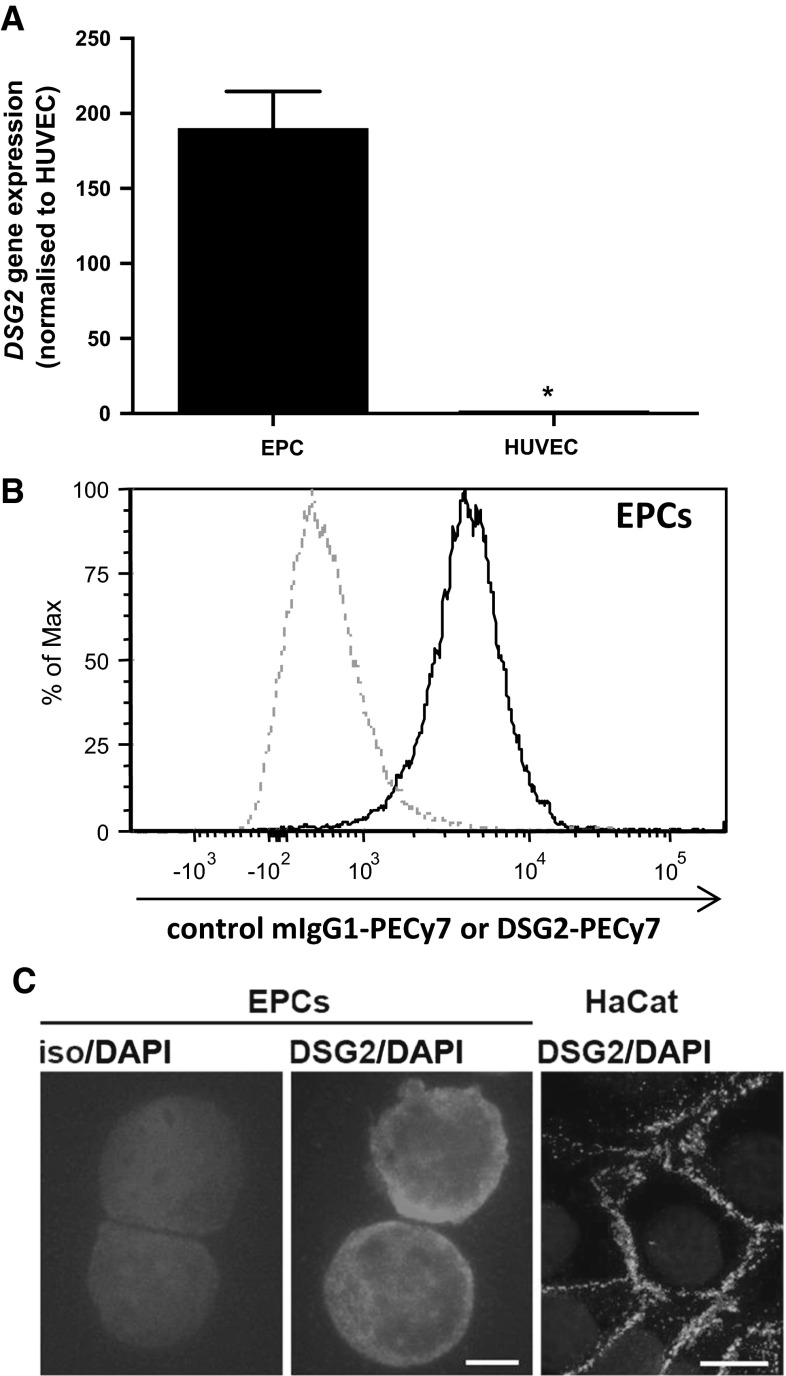
DSG2 expression by human naEFCs (EPCs). In **a**, DSG2 mRNA expression in UCB-derived CD133+ human naEFCs and HUVECs was determined by qPCR and expression values are shown as mean ± SEM, *n* = 3–4, * *p* < 0.05. In **b**, evaluation by flow cytometry of DSG2 protein expression (*solid line*) on EPCs. Dotted line shows isotype control. **c** Immunofluorescence evaluation of DSG2 protein expression on EPCs (*middle*, *scale bar* is 5 μm) and HaCat cells (*right*, *scale bar* is 20 μm). Isotype control (iso) staining on EPCs (*left*). EPC stainings in **b**–**c** are representative of results from ≥3 independent EPC donors

In a global comparative gene expression analysis of human umbilical cord blood (UCB)-derived CD133^+^CD34^+^VEGFR2^+^ non-adherent endothelial progenitor cells (EPCs) and donor matched HUVECs, we identified DSG2 to be significantly elevated in EPCs (NCBI Gene Expression Omnibus (GEO) Accession number GSE25979) [22]. Validation by qRT-PCR in additional donors confirmed that EPCs express ~180-fold more *DSG2* mRNA when compared to HUVEC (Fig. 1a). Subsequent flow cytometric analysis confirmed surface expression of DSG2 by EPCs (Fig. 1b) with immunofluorescence microscopy revealing a diffuse distribution of DSG2 over the cell surface and through the cytoplasm (Fig. 1c). This pattern of expression is unusual for desmosomal cadherins, which normally exhibit punctate distribution in desmosome-forming cells such as the HaCat keratinocytes (Fig. 1c).

**Fig. 2:**
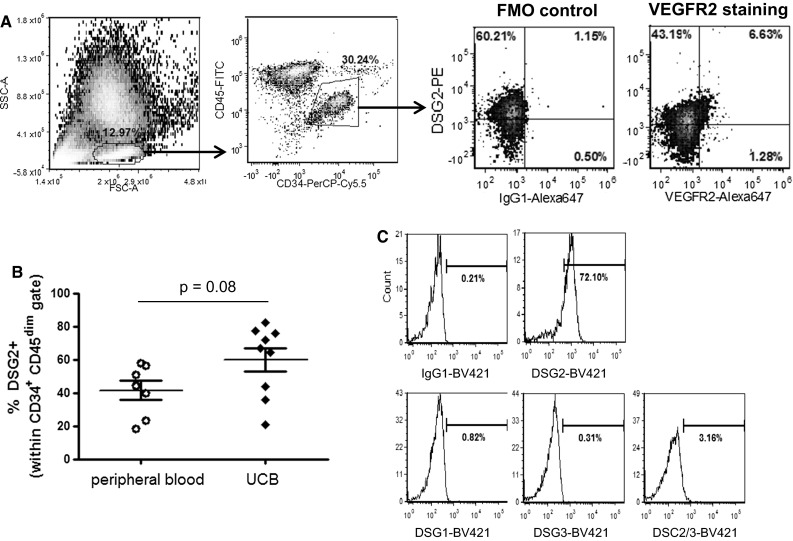
Unique expression of DSG2, but not other desmosome components, on circulating progenitor cells including EPCs. In **a**, lineage-depleted peripheral blood was analysed by flow cytometry for co-expression of DSG2 and VEGFR2. The gating strategy involved initial selection of cells based on physical parameters (FSC/SSC; left), followed by gating of the CD34^+^ CD45^dim^ progenitor population (*centre*), within which staining for DSG2 in combination with either control IgG (FMO [fluorescence-minus-one] control) or anti-VEGFR2 was determined. Results are representative of 4 donors. In **b**, the proportion of DSG2^+^ cells was compared for peripheral and umbilical cord blood samples, gating on CD34^+^ CD45^dim^ progenitor cells as shown above. **c** UCB samples were analysed by flow cytometry for expression of the indicated desmosomal cadherins, gating on CD34^+^CD45^dim^ progenitor cells, in comparison with isotype control. Results are representative of 3 donors

As these particular non-adherent EPCs are derived following 4 days of ex vivo culture [22], we also evaluated the expression of DSG2 on freshly isolated EPCs; that is, the small subpopulation of circulating CD34^+^ progenitor cells that co-express VEGFR2 [36, 37]. In progenitor-enriched peripheral blood from healthy donors, we used flow cytometric analysis to identify a small subset of VEGFR2^+^ cells (3.4 ± 1.4 %) within the CD34^+^ CD45^dim^ progenitor cell gate, and observed a high level of DSG2 expression within this EPC population (53.7 ± 13.6 % DSG2^+^; Fig. 2a). Interestingly, these experiments also revealed that DSG2 is expressed by VEGFR2-negative CD34^+^CD45^dim^ progenitor cells (41.6 ± 5.9 % DSG2^+^), the vast majority of which are expected to be hematopoietic stem and progenitor cells. Expression of DSG2 was also observed on CD34^+^ CD45^dim^ progenitor cells in freshly isolated UCB, with a trend towards higher expression compared to peripheral blood (Fig. 2b). Given that DSG2 typically combines with other desmosomal proteins [e.g., desmocollins (DSC1-3)] to form desmosomes between adjacent cells [4], we also examined expression of the four other desmosomal cadherins for which monoclonal antibodies are commercially available. As shown in Fig. 2c, we were unable to detect DSG1, DSG3, DSC2 or DSC3 (these latter two are recognised by the same mAb) on CD34^+^CD45^dim^ progenitor cells in UCB, although as expected, DSG2 was readily detectable. Further immunostaining revealed that CD34^+^CD45^dim^DSG2^+^ cells exhibited homogeneous expression of CD31 and high levels of CD117 and CD133 expression, but very little expression of the mature endothelial cell marker VE-cadherin (CD144) (Online resources Supp. Figure 1). These data demonstrate that circulating progenitor cells, including VEGFR2^+^ EPCs, express DSG2, but no other desmosomal cadherins, suggesting a role for DSG2 in these cells independent of desmosome function.

### DSG2 expression in the hematopoietic lineage is restricted to subsets of stem and progenitor cells

**Fig. 3:**
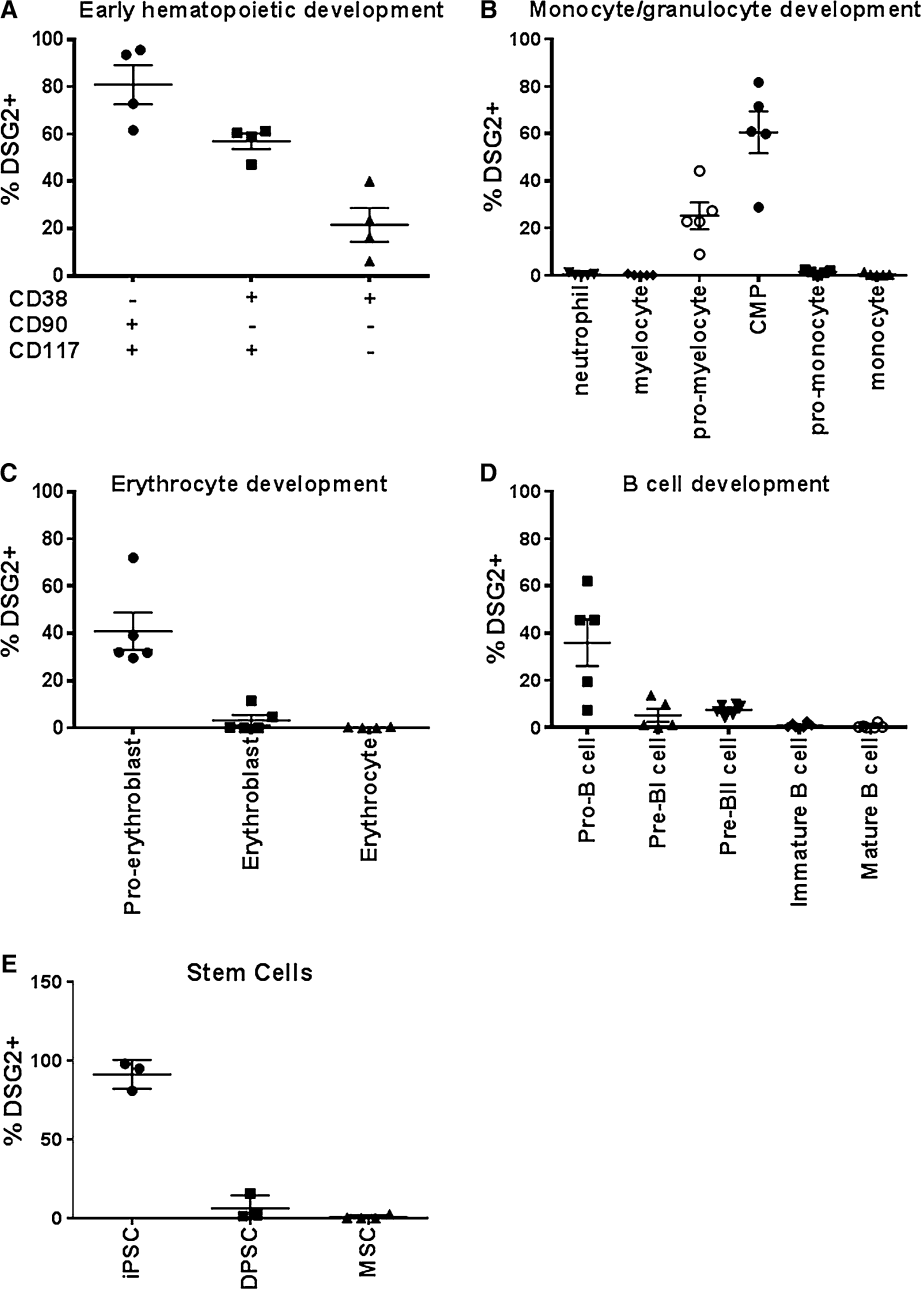
Unique expression of DSG2 on immature hematopoietic progenitor cells from human BM. Samples of normal human BM were subject to erythrocyte lysis and stained for flow cytometric analysis. In **a**, the expression of DSG2 in early hematopoietic progenitor cell development was examined in CD34+CD45dim progenitors by further gating on CD38−CD90+, CD38+CD90−CD117+ and CD38+CD90−CD117− subsets. In **b**, the expression of DSG2 in myeloid development was examined by gating on neutrophils (CD11b+CD13+CD33+CD16+CD117−); myelocytes (CD11b+CD13+CD33+CD16−CD117−); pro-myelocytes (CD34−CD117+CD45dimCD33+CD13+); CMP (common myeloid progenitors: CD34+CD117+CD45dimCD33+CD13+); pro-monocytes (CD11b+CD13+CD45dimCD14−) and mature monocytes (CD11b+CD13+CD33hiCD45brightCD14+). In **c**, the expression of DSG2 in erythrocyte development was examined by gating on pro-erythroblasts (CD34+CD45dimCD117+CD71+CD235a−); erythroblasts (CD34−CD45−CD117−CD71+CD235a+) and erythrocytes (CD34−CD45−CD117−CD71−CD235a+). In **d**, the expression of DSG2 in B cell development was examined by gating on pro-B cells (CD34+CD45dimCD22+CD19−CD10−CD20−); pre-BI cells (CD34+CD45dimCD22+CD19+CD10+CD20−); pre-BII cells (CD34−CD45dimCD22+CD19+CD10+CD20±); immature B cells (CD34−CD45brightCD22+CD19+CD10+CD20bright) and mature B cells (CD34−CD45brightCD22+CD19+CD10−CD20bright). In **e**, expression of DSG2 on iPS cells but not DPSC and MSCs was identified. Dots represent individual biological replicates, except for MSCs wherein a cell line was repeatedly tested

We showed in Fig. 2 that a large proportion of circulating CD34^+^CD45^dim^ cells express DSG2. While this population includes cells with EPC activity [18, 38], it also harbours major hematopoietic stem and progenitor cell subpopulations. To assess DSG2 expression in early hematopoietic development, normal human BM was stained with antibodies against the stem/progenitor markers CD34, CD38, CD90 and CD117. In CD34^+^CD45^dim^ human BM progenitors, DSG2 expression was highest and almost ubiquitous on CD38^−^CD90^+^ cells, which are thought to contain the most primitive and long-term repopulating hematopoietic stem cells [39] (Fig. 3a). A relative reduction in DSG2 expression was observed on CD38^+^CD90^−^CD117^+^ cells, with further reduction in CD38^+^CD90^−^CD117^−^ cells (Fig. 3a) indicating progressive loss of DSG2 expression with increasing lineage commitment.

Investigation of myeloid lineages revealed DSG2 expression on common myeloid progenitors (CMP; CD34^+^CD45^dim^CD117^+^CD33^+^CD13^+^) and on pro-myelocytes, in which CD34 is no longer expressed (CD34^−^CD45^dim^CD117^+^CD33^+^CD13^+^) (Fig. 3b). DSG2 was also observed on ~40 % of pro-erythrocytes (CD34^+^CD45^dim^CD117^+^CD71^+^CD235a^−^) and B cell lineage progenitors, including pro-B cells (CD34^+^CD45^dim^CD22^+^CD19^−^CD10^−^CD20^−^) (Fig. 3c, d).

For each of these lineages, DSG2 expression progressively decreased as a function of differentiation and was absent on all analysed mature cell types in the BM (Fig. 3b–d). This is in keeping with our observations of mature leucocyte populations in peripheral blood being negative for DSG2 (CD3^+^ T cells, CD14^+^ monocytes, CD16^+^ NK cells and CD19^+^ B cells) (Online resources Supp. Figure 2). Interrogation of publically available microarray data using the HemaExplorer tool [40] revealed near perfect concordance with our analysis at the gene expression level. Interestingly, further investigation revealed that *DSG2* gene expression correlates tightly with expression of *PROM1*, which encodes the CD133 progenitor marker (*R*^2^ = 0.92) as well as expression of *CD34* (*R*^2^ = 0.60).

Using flow cytometric analysis, we also examined expression of DSG2 on other stem cell populations and observed that DSG2 was strongly and uniformly expressed on three independently generated lines of induced pluripotent stem (iPS) cells (Fig. 3e). Interestingly, the parental fibroblast lines from which the iPS cells were derived completely lacked DSG2 expression (Online resources Supp. Figure 3). Also shown in Fig. 3e, three independently generated lines of dental pulp stem cells (DPSCs) and repeated measures of a MSC-like line demonstrated that DSG2 was not expressed at detectable levels by these progenitor cell types. Thus, DSG2 is expressed by pluripotent stem cells but not by stem cells with restricted mesenchymal differentiation potential.

### DSG2^+^ progenitor cells expand in vitro and exhibit multi-potent differentiation potential

**Fig. 4:**
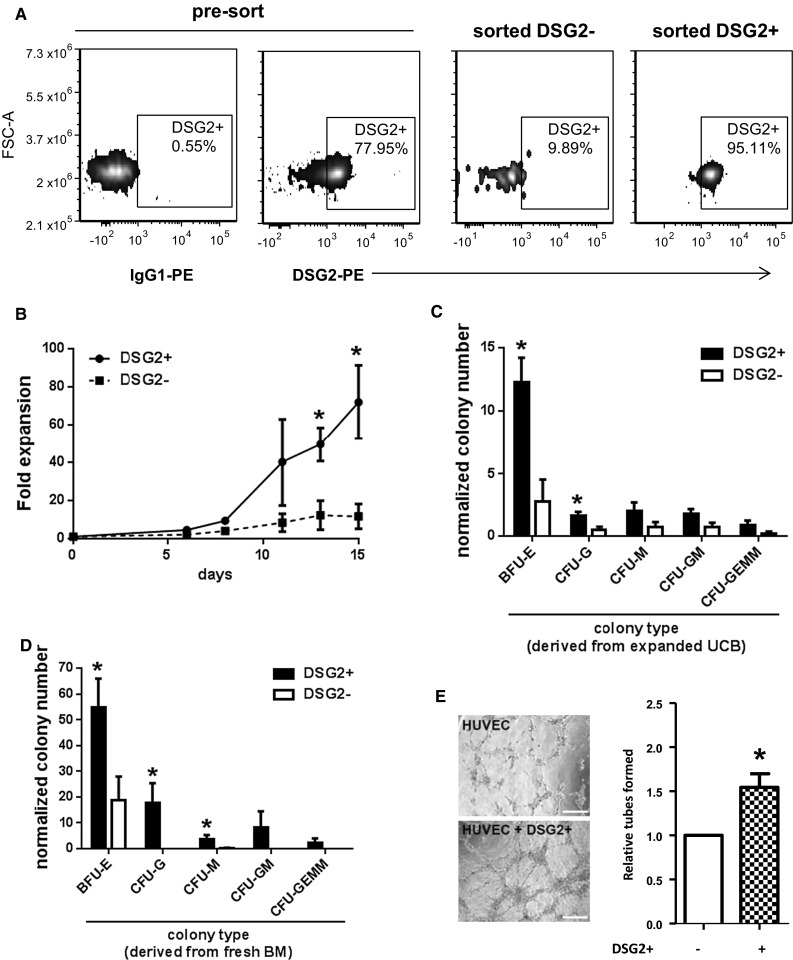
Enrichment of DSG2+ progenitor cells demonstrate a pro-proliferative and multi-potential nature. In **a**, samples of UCB were sorted for DSG2+ and DSG2− subsets. Pre-sorted CD34+CD45dim-gated cells were stained for IgG control (*far left panel*) and DSG2 (*middle left panel*), and compared to the DSG2 staining amongst post-sort DSG2+ (*middle right panel*) and DSG2− (*far right panel*) cells. In **b**, DSG2+ and DSG2− cell expansion was calculated over 15 days. Results are mean ± SEM, *n* = 3 donors, *p < 0.05. In **c**, expanded UCB-derived DSG2+ and DSG2− cells and in **d**, freshly sorted BM-derived DSG2+ and DSG2− cells were seeded for colony formation. Cells formed blast-forming unit-erythroid (BFU-E), colony-forming units (CFU)-GEMM, -GM, -G and -M colonies. Results are mean ± SEM, *n* = 3–4 donors, *p < 0.05 compared to DSG2− cells. In **e**, a representative image of the Matrigel angiogenesis assay performed with HUVEC alone (upper image) or HUVEC co-cultured with DSG2+ sorted and expanded UCB cells (lower image) in vitro. Relative tube-like structure numbers formed in each condition from 3 independent experiments are quantified on the right as mean ± SEM, *p < 0.05 versus HUVEC alone. *Scale bar* is 20 μm

To assess the functional capability of circulating DSG2^+^ progenitor cells, we isolated CD34^+^CD45^dim^DSG2^+^ cells and CD34^+^CD45^dim^DSG2^−^ cells from UCB MNCs via cell sorting. The purities of sorted cells were 97 ± 1 % for DSG2^+^ cells (Fig. 4a) and 89 ± 4 % for DSG2^−^ cells. Due to the low numbers (<1 × 10^4^) of cells isolated in these experiments, cells were expanded for 15 days in defined growth medium [29] prior to further investigation. While both DSG2^−^ and DSG2^+^ populations expanded during culture, expansion was significantly greater in DSG2^+^ population at several time-points (Fig. 4b).

We next conducted cell fate analyses of the expanded DSG2^+^ versus DSG2^−^ cell populations using methylcellulose hematopoietic colony formation assays. DSG2^+^ cells cultured with GM-CSF, SCF, IL-3 and EPO for 14 days produced various colony types, including bi-potent progenitors of the erythrocyte and myeloid lineages (CFU-GEMM) and erythrocyte lineage (BFU-E) and granulocyte/monocyte lineage (CFU-GM, CFU-G and CFU-M) progenitors (Fig. 4c). Notably, the DSG2^+^ cells generated substantially more BFU-E and CFU-G colonies compared to DSG2^−^ cells (Fig. 4c). Similarly, freshly isolated, unexpanded DSG2^+^ cells purified from fresh human adult BM gave rise to significantly more BFU-E, CFU-G and CFU-M colonies compared with freshly isolated, unexpanded DSG2^−^ cells (Fig. 4d). These findings suggest that CD34^+^CD45^dim^DSG2^+^ hematopoietic progenitor cells have multi-lineage differentiation potential and enhanced colony formation capacity compared to their DSG2^−^ counterparts.

We next examined the capacity of the DSG2^+^ sorted cells to promote endothelial tube-like formation using Matrigel [22, 29]. HUVEC were seeded either alone or after mixing with expanded DSG2^+^ cells, and the formation of capillary-like networks was monitored over a 6 h period. Quantitation of vessel formation identified a significant increase in the number of tube-like structures from HUVEC in the presence of DSG2^+^ cells, suggesting that DSG2^+^ cells are pro-angiogenic (Fig. 4e). As expected from their immature phenotype, DSG2^+^ cells were unable to form tubes in the absence of HUVEC (data not shown).

### Heterogeneous expression of DSG2 in mature ECs in vivo

**Fig. 5:**
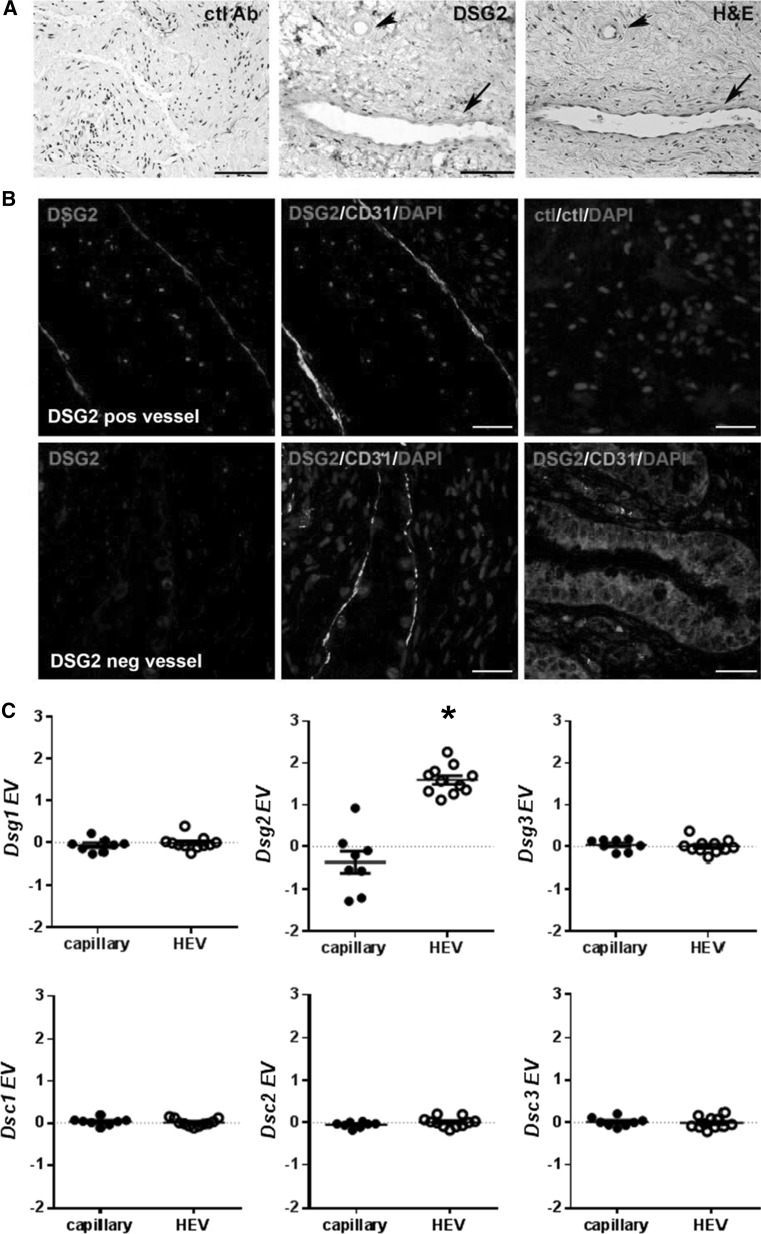
Mature ECs display heterogeneous expression of DSG2. In **a**, representative IHC images of human cervical tissue with positive brown staining for DSG2 (*centre panel*) wherein an arteriole (*arrow head*) and a venule (*arrow*) are shown. The same tissue area is also shown stained separately with H&E, allowing visualisation of vessel architecture (*right panel*). Isotype control staining of cervical tissue (*left panel*). *Scale bar* is 0.2 mm. In **b**, representative immunofluorescence images of human cervical tissue with DSG2 (*red*), CD31 (*green*) and DAPI (*blue*) illustrate a DSG2+ vessel (*top left* and *middle*) and a DSG2^−^ vessel (*bottom left* and *middle*). Staining controls on the right show the same cervical tissue with relevant isotype controls (*top right*; *negative control*) and cervical epithelium (*bottom right*; *positive control*). *Scale bar* is 30 μm. In **c**, gene expression values (EV) of *Dsg1*–*3* and *Dsc1*–*3* mined from GEO dataset GSE58056. *Each symbol* represents an individual biological replicate from capillary or high endothelial venules (HEV); *small horizontal lines* indicate the mean, *p < 0.05 versus capillary. (Color figure online)

Results thus far suggest that DSG2 is expressed by freshly isolated as well as short-term-cultured EPCs and that DSG2^+^ progenitor cells have pro-angiogenic activity in vitro. Based on these findings, we examined whether DSG2^+^ cells were present in mature endothelium. To address this, a human tissue microarray with multiple cores of 30 normal and 38 cancerous tissues was examined morphologically for the presence of DSG2 on red blood cell-containing lumenised blood vessels. We identified vessels in 16 normal tissues and 13 tumours (Online resources Supp. Table 2). For the normal tissues, 8/16 (50 %) contained vessels with DSG2^+^ ECs in at least one of three stained sections per tissue, while all vessels in the other tissues lacked detectable DSG2 expression. In cancerous tissues, 7/13 tumour specimens (54 %) contained DSG2^+^ vessels (Online resources Supp. Table 2). To note, immunohistochemistry on human cervix tissue from a healthy individual revealed that DSG2 can be expressed by the endothelium of both arterioles (Fig. 5a, arrow head) and venules (Fig. 5a, arrow). To further confirm the expression of DSG2 by vascular ECs, multi-colour immunofluorescence microscopy was performed after co-staining sections for DSG2 and CD31. This identified heterogeneous expression of DSG2 in ECs in normal human cervix, with both CD31^+^DSG2^+^ (Fig. 5b, top left and top middle panels) and CD31^+^DSG2^−^ (Fig. 5b, bottom left and bottom middle panels) vessels evident.

In silico analysis of publically available microarray data revealed that *Dsg2* is also heterogeneously expressed by the mouse vasculature. In a recent study, Lee and colleagues generated whole genome expression data from lymphoid tissue-derived ECs isolated from either high endothelial venules (HEV) or capillaries (GSE58056) [41]. Our interrogation of this data using GEO2R revealed significantly elevated levels of *Dsg2* in the HEV-derived ECs compared to capillary-derived ECs from the lymphoid tissue (Fig. 5c). No difference in expression was observed for other desmosomal cadherins (*Dsg1&3* and *Dsc1*-*3*) (Fig. 5c).

### Generation of *Dsg2*^lo/lo^ mice

**Fig. 6:**
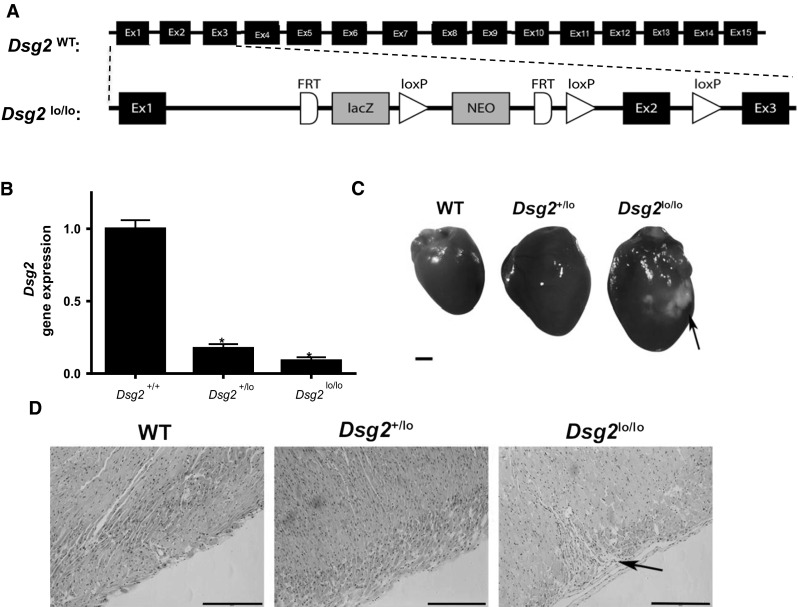
Disruption of intron 1 of *Dsg2* results in a hypomorphic DSG2 allele. In **a**, a *lacZ*/neomycin cassette was incorporated into intron 1 of mouse *Dsg2* by targeted insertion. **b** The resulting *Dsg2*-LacZ-neomycin allele exhibited reduced *Dsg2* expression in the mouse hearts of WT, *Dsg2* ^+/lo^ and *Dsg2* ^lo/lo^ mice by quantitative PCR (qPCR). Results are mean ± SEM, *n* = 3, *p < 0.05 versus WT. In **c,** morphological comparison of hearts from WT, DSG2^+/lo^ and *Dsg2* ^lo/lo^ mice with arrows identifying fibrotic lesions. *Scale bar* is 3 mm. In **d,** dissected hearts stained with hematoxylin/eosin and an arrow identifying fibrotic lesions. *Scale bar* is 0.5 mm

To study DSG2 function in vivo, we generated a ‘knockout-first’ *Dsg2* mouse strain on a C57BL/6 N background. This ‘gene trap’ approach utilised a targeted insertion of an FRT-flanked lacZ-neomycin cassette into intron 1 of the murine *Dsg2* locus (see schematic of the inserted cassette in Fig. 6a). Mice heterozygous for insertion of the *lacZ*-neomycin construct were inter-bred and yielded fertile wildtype (WT), heterozygous and homozygous offspring at the expected Mendelian ratios (1:2:1), with litter sizes similar to WT C57BL/6N controls.

Upon examination of *Dsg2* gene expression in these mice, we observed a significant reduction in *Dsg2* mRNA in the heart (where *Dsg2* is strongly expressed [42]) in heterozygous and homozygous mice compared to WT mice (Fig. 6b), indicating perturbed *Dsg2* transcription with this ‘knockout-first gene trap’ approach. Similar results were observed in the skin (data not shown). This observation of reduced *Dsg2* expression in the heart prompted us to examine for cardiac fibrosis, a previously reported phenotype of *Dsg2*-knockout mice [5, 43] and humans with *DSG2* mutations [3, 6]. In dissected hearts of the homozygous mutant mice, fibrotic lesions were frequently seen in the enlarged hearts (Fig. 6c, arrow). Fibrotic lesions were confirmed by histological analysis using a hematoxylin/eosin stain with an arrow identifying a fibrotic lesion (Fig. 6d). Notably, this pathology was less evident in the heterozygous mice (Fig. 6c, d). We thus concluded that we had generated a ‘hypomorphic’ *Dsg2* allele (termed *Dsg2*^lo^) which resulted in normal viability and fertility and is in contrast to the embryonic lethality observed in the *Dsg2*-knockout mice detailed in Eshkind et al. [44]. Based on these observations, we reasoned that our *Dsg2* line would provide a useful tool for evaluating the in vivo consequences of deficiency.

### A role for DSG2 in neoangiogenesis

**Fig. 7:**
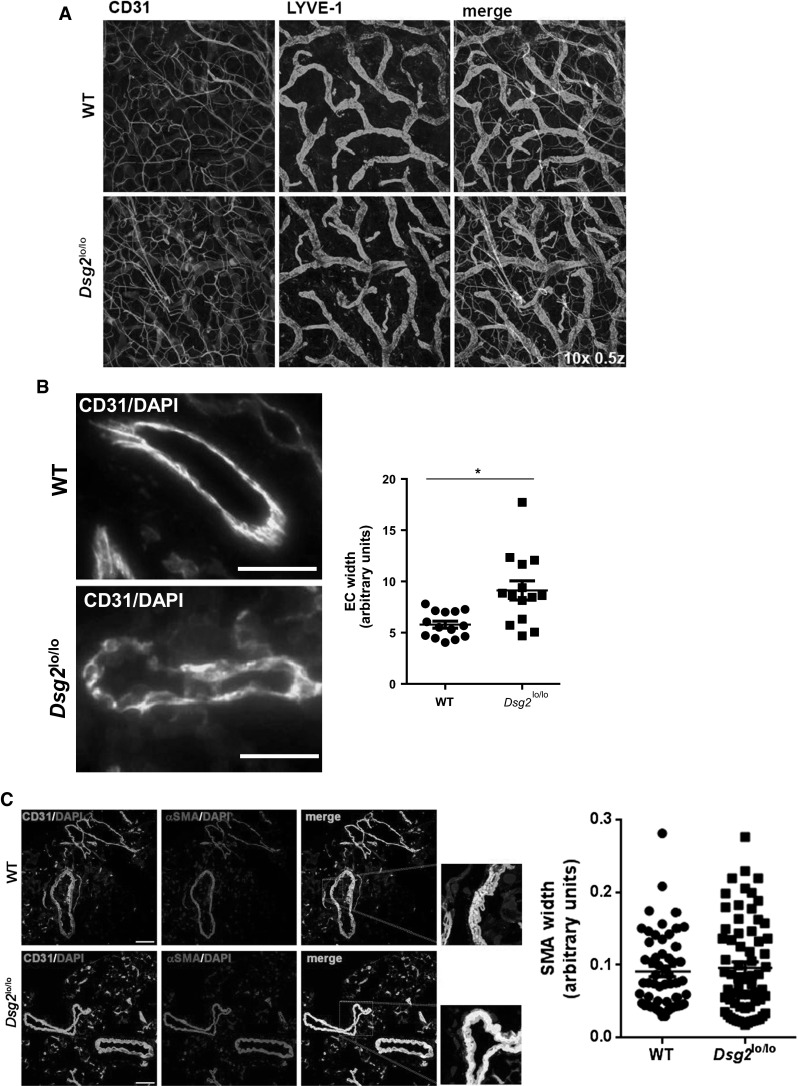
*Dsg2* ^lo/lo^ mice exhibit altered EC morphology. In **a,** a representative image of blood (CD31, *green*) and lymphatic (LYVE-1, *blue*) vessels in mid-ear sections of WT and *Dsg2* ^lo/lo^ adult mice with αSMA shown in the merged image (*red*). In **b**, representative fluorescence images of CD31^+^ ECs (*yellow*) in pancreatic sections of control (WT) and *Dsg2* ^lo/lo^ mice, with DAPI nuclei staining (*blue*), *scale bar* is 50 μm. The *graph* shows analysis of EC width (measured in 6 positions per vessel) from five vessels per mouse. Results are mean ± SEM, *n* = 3 mice/group, *p < 0.05 versus WT. In **c**, representative fluorescence images of pancreatic sections of control (WT) and *Dsg2* ^lo/lo^ mice, with CD31^+^ ECs (*green*), αSMA perivascular cells (*red*) and DAPI nuclei staining (*blue*). *Scale bar* is 0.1 mm. The *graph* shows analysis of vessel width (measured in 6 positions per vessel) from five vessels per mouse. Results are mean ± SEM, *n* = 4 mice/group. (Color figure online)

A gross comparison of the development and patterning of blood and lymphatic vessels in adult skin was then conducted in the ears of adult WT and *Dsg2*^lo/lo^ mice via whole mount immunostaining using markers to blood (CD31^+^) and lymphatic (CD31^+^ LYVE-1^+^) vascular ECs. As shown in Fig. 7a, no overt differences in the macroscopic patterning of either vessel type were observed in *Dsg2*^lo/lo^ mice. However, microscopic examination of the ECs within a cross section of pancreatic vessels suggested otherwise. As shown in Fig. 7b, high magnification images revealed morphological abnormalities of CD31^+^ ECs in the *Dsg2*^lo/lo^ mice, including regions of increased junctional hypertrophy and an undulating luminal surface. Analysis of EC width using Image J revealed that the vascular wall width of the *Dsg2*^lo/lo^ mice was significantly greater than that of the control mice. To visualise perivascular cells associated with the blood vasculature, we examined αSMA expression using immunohistochemistry. Image J analysis of the SMA staining patterns failed to detect any differences between the WT and *Dsg2*^lo/lo^ mice, suggesting no alteration in vessel contractility (Fig. 7c).

**Fig. 8:**
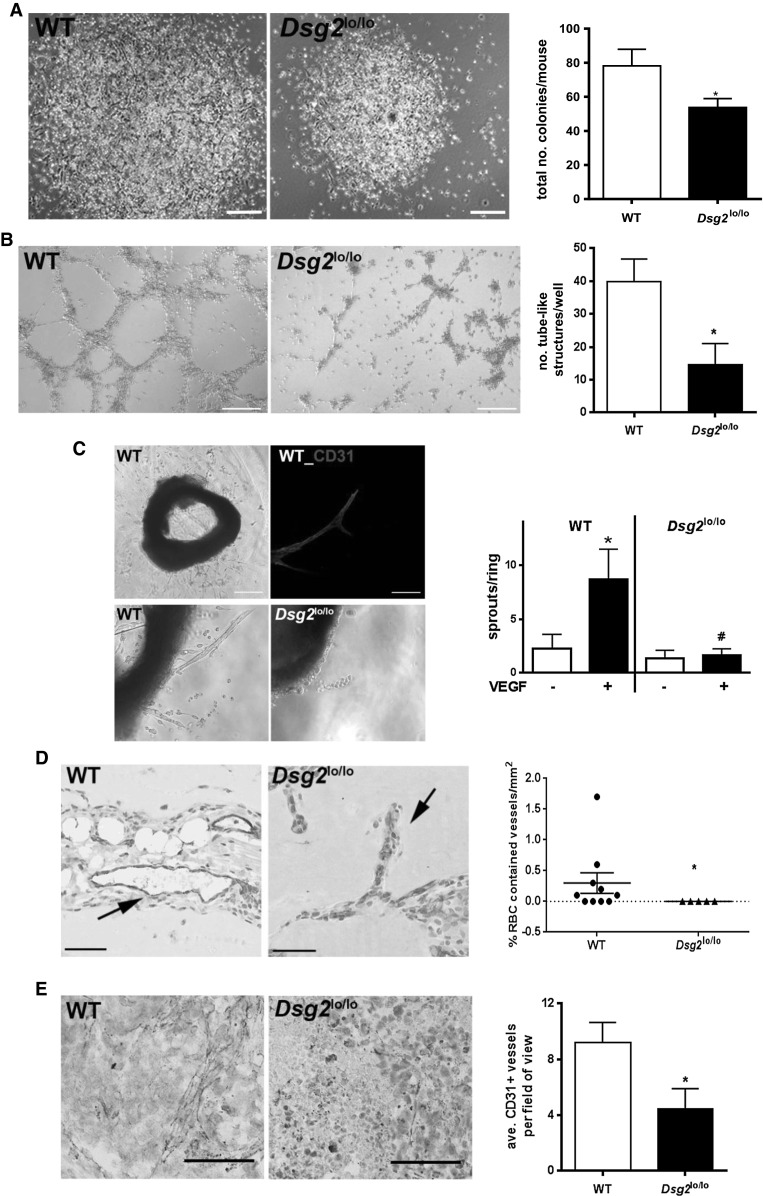
*Dsg2* ^lo/lo^ mice exhibit reduced vascular development. In **a**, BM-derived EC colonies were generated from WT and *Dsg2* ^lo/lo^ mice by culture on fibronectin plates for 6 days. Images on the *left* show representative colonies, while the graph on the right depicts the number of colonies per well (mean ± SEM, *n* = 3, *p < 0.05 versus WT, *scale bar* is 0.2 mm). **b** shows BM-derived ECs from WT and *Dsg2* ^lo/lo^ mice seeded onto Matrigel. Tube-like structure formation captured by phase-contrast microscopy 4 h post-seeding. Results are mean ± SEM, *n* = 3, *p < 0.05 versus WT. *Scale bar* is 0.2 mm. In **c**, phase-contrast images of aortic rings embedded in Matrigel showing microvessel outgrowth in response to VEGF (*top left*, *scale bar* is 0.5 mm); CD31 staining of the vascular sprouts (*top right*, *scale bar* is 0.1 mm); and sprouts counted 5 days post embedding. Higher magnification of rings shown for WT (*bottom left*) and *Dsg2* ^lo/lo^ (*bottom right*). Results are mean ± SEM, *n* = 3, *p < 0.05 versus no VEGF, # *p* < 0.05 versus WT + VEGF. In **d**, Matrigel plugs in WT and *Dsg2* ^lo/lo^ mice were retrieved and stained with anti-CD31 (*brown*) and hematoxylin (*lilac*). *Scale bar* is 50 μm. The number of erythrocyte-containing vessels per mm^2^ was quantified. Data expressed as mean ± SEM, *n* = 5–10, *p < 0.05 versus WT. In **e**, B16 melanomas in WT and *Dsg2* ^lo/lo^ mice were retrieved and stained with anti-CD31 (*red*) and eosin (*blue*). *Scale bar* is 100 μm. The average number of CD31^+^ vessels per field of view was quantified. Data expressed as mean ± SEM, *n* = 7, *p < 0.05 versus WT (Mann–Whitney). (Color figure online)

As CD34^+^CD45^dim^DSG2^+^ cells displayed a pro-angiogenic phenotype in vitro (Fig. 4e), we interrogated this further using ECs obtained from *Dsg2*^lo/lo^ mice. First, we compared the ability of BM-derived EPCs from WT and *Dsg2*^lo/lo^ mice to form endothelial colonies on fibronectin-coated culture dishes [32]. As shown in Fig. 8a, BM-derived ECs from *Dsg2*^lo/lo^ mice were smaller in colony size and lower in total colony number when compared to WT controls. Consistent with this, BM-derived ECs from *Dsg2*^lo/lo^ mice showed reduced formation of tube-like structures on Matrigel compared with BM-derived ECs from WT mice (Fig. 8b). We also compared WT and *Dsg2*^lo/lo^ mice for their ability to generate endothelial sprouts in an aortic ring assay [33]. The top left image of Fig. 8c demonstrates that aortic rings from WT mice generate sprouts in response to VEGF, and the top right image of Fig. 8c confirmed that the sprouts were CD31^+^ ECs. Notably, the lower panels of Fig. 8c and the quantified data in the graph illustrate that *Dsg2*^lo/lo^ mice produced significantly fewer vascular sprouts in response to VEGF compared to aortic rings from WT mice. Next, neoangiogenesis was tested in vivo by injection of a Matrigel bolus into the flanks of WT and *Dsg2*^lo/lo^ mice, followed 7 days later by immunohistochemical examination of excised plugs for evidence of angiogenic sprouting. While plugs from WT mice contained numerous erythrocyte-containing CD31^+^ vessels, these were largely absent in plugs from *Dsg2*^lo/lo^ mice (Fig. 8d). Finally, we translated this into a more clinically relevant in vivo model by examining the vasculature of B16 melanomas in the WT and *Dsg2*^lo/lo^ mice. At approximately 20 days post-injection, immunohistochemical examination of excised tumours showed that tumours from WT mice contained significantly more CD31^+^ vessels than the tumours in the *Dsg2*^lo/lo^ mice (Fig. 8e).

### Knockdown of DSG2 in human ECs suggests a role in EC barrier integrity, adhesion and migration

**Fig. 9:**
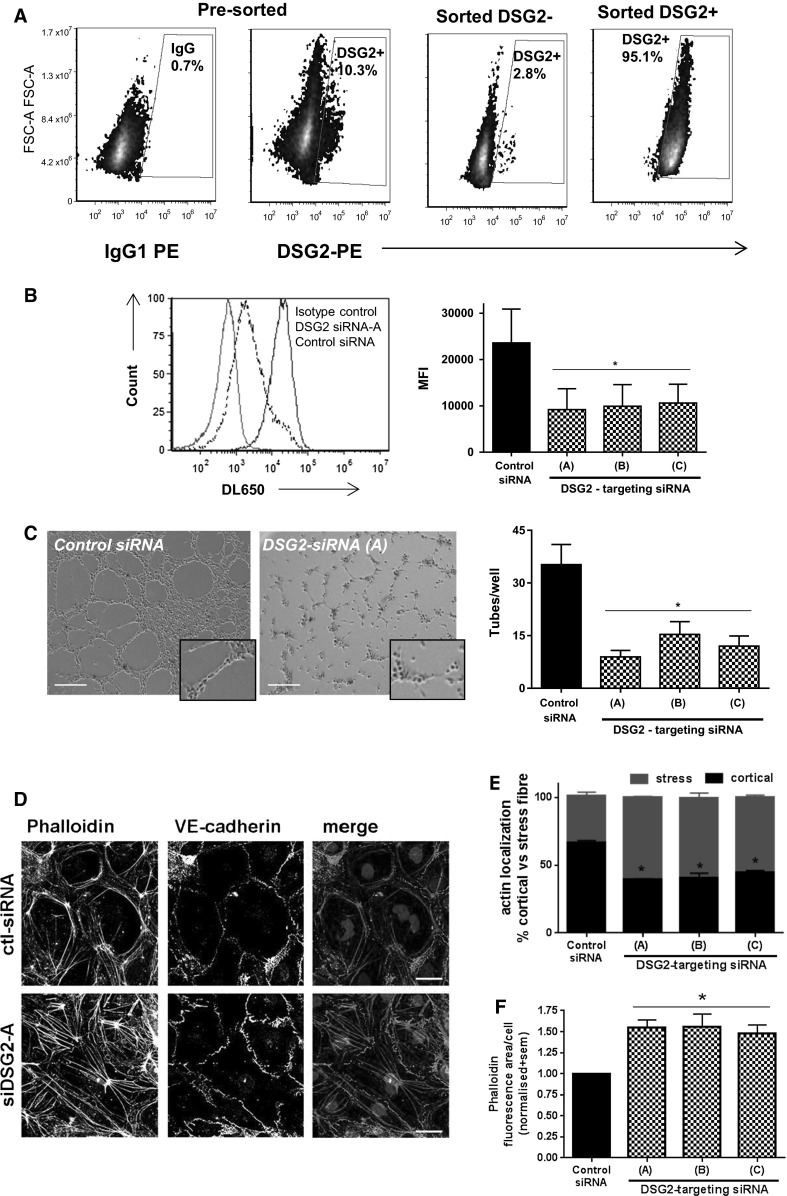
DSG2 knockdown alters BMEC function and cytoskeletal composition. In **a**, BMEC cells were sorted for DSG2^+^ and DSG2^−^ subgroups. Results show pre-sort cells stained for control IgG (*far left panel*) or DSG2 (*middle left panel*), and compared to the DSG2 staining amongst post-sort DSG2+(*far right panel*) and DSG2^−^ (*middle right panel*) cells. In **b,** the sorted DSG2+BMEC cells were subject to *DSG2* knockdown using siRNA and analysed for DSG2 protein expression via flow cytometry after 72 h. Histogram shows staining with control IgG (*red*), or DSG2 after treatment with non-targeting control siRNA (*blue*) or *DSG2*-targeting siRNA-A (*dotted black*). The graph shows DSG2 expression quantified as mean fluorescence intensity (MFI). In **c**, representative images of the effect of DSG2 knockdown on tube-like structure formation by sorted DSG2 + BMEC cells after 6 h on Matrigel are shown on the *left* (*scale bar* is 0.2 mm), while the number of tube-like structures formed per well was quantified on the right. Data expressed as mean ± SEM, *n* = 6, *p < 0.05 versus control siRNA. In **d**, representative images of the effect of DSG2 knockdown on the localisation of actin (phalloidin, *far left*) and VE-cadherin (*middle*) and merged image (*far right* (phalloidin (*red*) and VE-cadherin (*green*)) are shown. *Scale bar* is 20 μm. In **e**, stratification of actin localisation via phalloidin staining of cortical or stress fibre formation for individual cells (defined by DAPI-stained nucleus) and normalized to control siRNA cells within an experiment. Data expressed as mean ± SEM, *n* = 3, *p < 0.05 versus control siRNA. In **f**, ImageJ was used to quantify the area of phalloidin staining relative to the number of nuclei per field of view and normalized to control siRNA cells within an experiment. Data expressed as mean ± SEM, *n* = 3, *p < 0.05 versus control siRNA. (Color figure online)

Using flow cytometry, we identified DSG2 expression in a subpopulation of cells present in the human BM-derived EC line TrHBMEC (BMEC) [45] (Fig. 9a). To investigate a role for DSG2 in EC function, we sorted for this DSG2^+^ subpopulation (Fig. 9a), resulting in a culture which was 95 % DSG2^+^ immediately after sorting and maintained similar levels of DSG2 expression for several passages. *DSG2* gene knockdown was performed on these cells, resulting in significant reductions in DSG2 surface protein using three different siRNA constructs (Fig. 9b). Using annexin V, 7-AAD and flow cytometry, we compared BMEC cell viability 72 h post-knockdown of *DSG2* and observed a similar percentage survival rate of 84 ± 5 for the control siRNA cells compared to 74 ± 6, 87 ± 3 and 79 ± 4 for siRNA-A, siRNA-B and siRNA-C, respectively. Next, we tested the function of DSG2-reduced BMEC cells in a Matrigel angiogenesis assay and observed a significant reduction in the number of tube-like structures for all three DSG2-targeting siRNA constructs when compared to the cells transfected with control siRNA. Figure 9c illustrates a representative image of tube-like structures formed as well as the quantitation from three separate experiments. Interestingly, close examination of the cells in this angiogenesis assay suggested that depletion of DSG2 prevented them from elongating, as they remained round in shape (Fig. 9c, enlarged insert). As actin filaments of the cytoskeleton are important components which control dynamic processes such as cell shape, cell–cell adhesion and cell migration (reviewed in [46]), we next investigated whether alteration of DSG2 affected actin assembly in ECs. Using the aforementioned siRNA knockdown approach and immunofluorescence staining for VE-cadherin to define cell borders, we observed that the actin filament-containing structures differed significantly when DSG2 was reduced. As shown in Fig. 9d, control cells showed junction-associated circumferential phalloidin stained actin filaments (cortical actin), while the DSG2-reduced BMEC cells exhibited cytoplasmic stress fibres. Quantitation of the amount of actin localised to the cell boundary (i.e., cortical) versus that covering a cell (i.e., in stress fibre formation) revealed that for all three DSG2-targeting siRNA constructs, a significant increase in stress fibre content was observed (Fig. 9e). Further examination with ImageJ confirmed that DSG2 knockdown significantly increased actin assembly into stress fibre formation, as reflected by an increase in the total area of phalloidin staining (Fig. 9f).

**Fig. 10:**
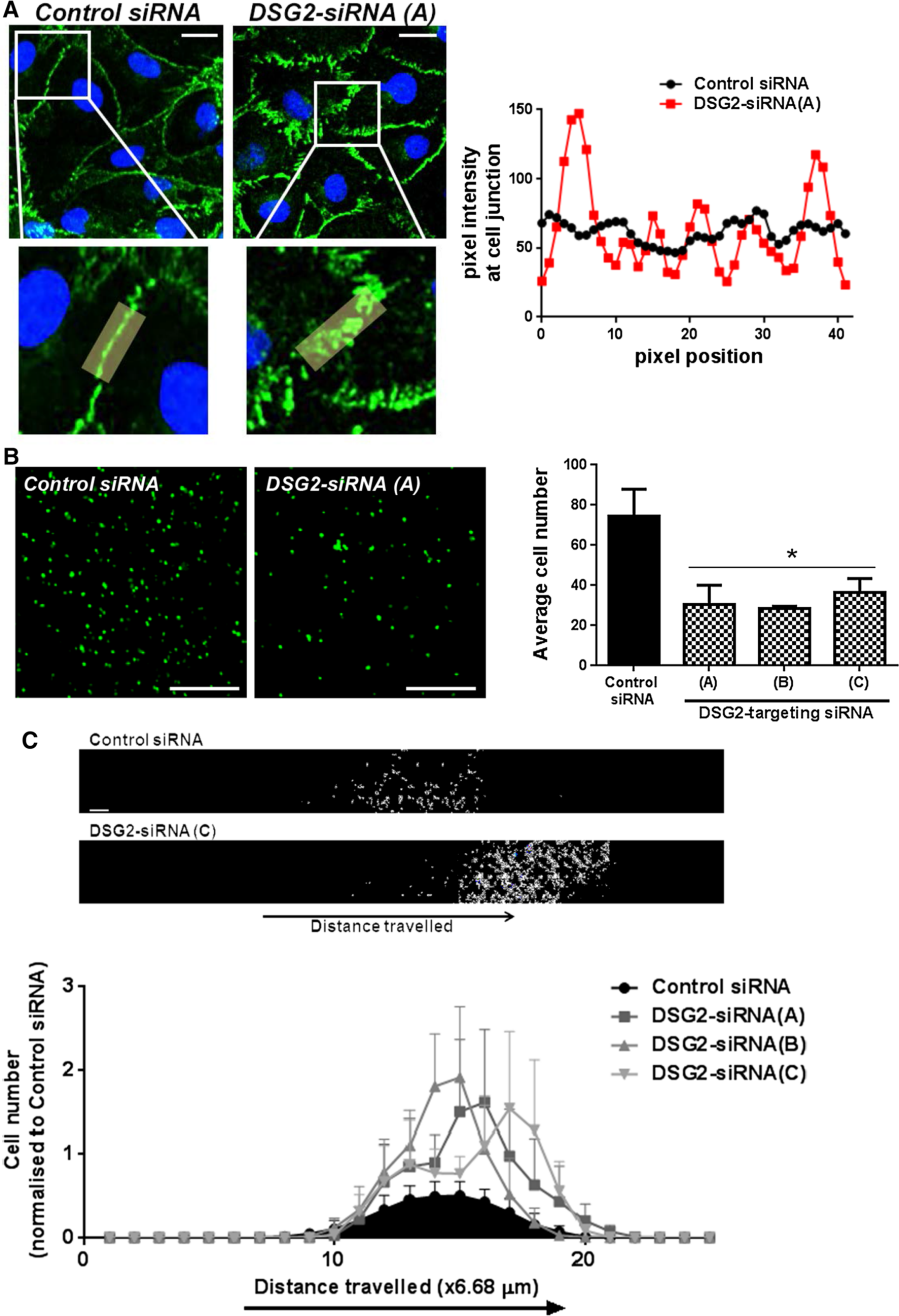
DSG2 knockdown alters BMEC cell junction formation, adhesion and invasion. In **a**, representative images of the effect of DSG2 knockdown on the localisation of VE-cadherin (*green*) with DAPI-stained nuclei (*blue*) are shown. *Scale bar* is 20 μm. Accompanying fluorescence intensities along the depicted lines depict localisation of VE-cadherin along a cell–cell junction. In **b**, the adhesion of fluorescently labelled BMEC cells to a confluent monolayer of unlabelled BMEC cells was determined using sorted DSG2 + BMEC cells treated with the indicated siRNA. Representative fluorescent images are shown on the *left* (*scale bar* is 0.2 mm), while the number of adherent cells per field of view was quantified on the right. Results are shown as mean ± SEM, *n* = 3–5, *p < 0.05 versus control siRNA. In **c**, BMEC treated with control or DSG2-targeting siRNA for 24 h were allowed to invade Matrigel covered Transwells in an inverse invasion assay. *Scale bar* is 0.4 mm. After four days of invasion, cells were stained with propridium iodide and serial optical sections (6.68-μm intervals) were acquired. Magnified images from *z*  =  22 sections are shown (*top*). Cell invasion was quantified as the number of cells over distance travelled and then normalised to peak control siRNA values for each experiment. Data show mean  ±  SEM, *n* = 6, *p* <  0.05, ANOVA. (Color figure online)

As stress fibre formation is often accompanied by actin-mediated contraction and remodelling of the VE-cadherin complex from a continuous to an interrupted patterning [47, 48], we next investigated whether VE-cadherin localisation was also affected by *DSG2* knockdown. Figure 10a shows that in control cells VE-cadherin is tightly maintained within the adherens junctions, while knockdown of *DSG2* reorients VE-cadherin into disjointed and perpendicular adhesions. Quantitation of VE-cadherin fluorescence intensities along cell junction lines (depicted by the opaque box overlay the enlarged images of Fig. 10a) supported this observation with VE-cadherin in control cells contained within the termini of short bundles while in the DSG2 knockdown cells was irregular in intensity. These observations, together with those in the aortic ring assay (Fig. 8c), prompted us to test the cell–cell interactions in an adhesion assay. Briefly, BMEC cells transfected with control or DSG2-targeting siRNAs were seeded into two culture dishes, and after 72 h one dish was trypsinised into a single-cell suspension and stained with calcein-AM, while the second remained as a confluent monolayer. The calcein-labelled BMEC (±DSG2-siRNA) were incubated with the BMEC monolayers (also ±DSG2-siRNA) for 15 min to permit adhesion prior to stringent washing to remove any unbound cells. The left panel of Fig. 10b shows a representative image of calcein-labelled adherent BMEC cells (±DSG2-siRNA) using fluorescence microscopy, while the graph shows quantitation from three separate experiments wherein a significant reduction in bound fluorescent cells following *DSG2* knockdown was observed. This data therefore suggest that DSG2 plays a critical role in regulating cell–cell adhesion between human mature ECs. To investigate whether DSG2 contributed to EC invasion, we conducted inverse invasion assays wherein BMECs (±DSG2-siRNA) penetrated through an extracellular matrix (Matrigel) towards a chemoattractant (VEGF). Interestingly, BMEC with reduced DSG2 exhibited greater invasive potential by being able to migrate further through the Matrigel matrix and towards VEGF (Fig. 10c, representative image). Quantitation of cell number relative to distance travelled confirmed that all three DSG2-targeting siRNA constructs significantly enhanced the number of cells invading into the Matrigel as well as their distance travelled (Fig. 10c).

## Discussion

This study reveals that, in addition to epithelial cells and cardiomyocytes, DSG2 is also expressed on sub-populations of human blood and BM-derived progenitor cells. We also provide multiple lines of evidence that DSG2 is expressed by cells of the endothelial lineage and that it contributes to neoangiogenesis. Our findings provide critical new information that DSG2 is much more than a simple desmosomal adhesion molecule.

In their canonical role, the Ca2^+^-binding transmembrane glycoprotein desmosomal cadherins DSG1-4 and DSC1-3 associate to form homotypic and heterotypic interactions with adjacent cells, resulting in specialised junctions designed to withstand mechanical stress in the myocardium and stratified epithelium [3, 4]. Here we have uncovered an additional role for DSG2, in non-desmosome-forming progenitor cells and endothelial cells. We first identified DSG2 as a highly expressed gene and cell surface protein on human EPCs. Further investigation revealed that DSG2 is also expressed on the closely related hematopoietic progenitor cell lineage, being detectable on early hematopoietic progenitors, common myeloid progenitors, pro-erythroblasts and pro-B-cells, but not on the more differentiated progenitors or mature leucocyte subsets. Our observation of a tight correlation between *DSG2*, *CD133* and *CD34* genes suggests that DSG2 may play an important, as yet unidentified, role in symmetric cell division and progenitor renewal. CD34 is the classical marker of hematopoietic progenitors and contributes to cell adhesion and hematopoietic differentiation [49]. BM-derived CD34^+^ cells reconstitute hematopoietic cells [50] and CD34 is also expressed by EPCs and mature ECs [22, 51]. CD133 is also a marker of hematopoietic stem and progenitor cells (HSPCs) and is restricted to ~20–60 % of the CD34^+^ cells isolated from adult BM, UCB or peripheral blood [52], and the CD133^+^CD34^+^ fraction of HSPCs has a high capacity for expansion [53]. We demonstrate that DSG2 is expressed almost ubiquitously by CD133^+^ cells and at highest levels by the CD38^−^CD90^+^ subset. Thus, our study identifies DSG2 as a new cell surface marker for the most primitive and proliferative of HSPCs. In data also shown here, we observed that DSG2 is expressed by induced pluripotent (iPS) cells, which supports our contention of this cadherin being a marker of progenitor cells and extends a previous publication of *DSG2* being one of the early activated genes in the reprogramming of fibroblasts to iPS cells [54]. Based on these findings, we propose that DSG2 is a new, and potentially useful, marker of primitive HSPCs and we speculate that it may influence their proliferation and survival. One intriguing possibility is that DSG2 on the surface of HSPCs undergoes homotypic interactions with DSG2 expressed by ECs within the BM, thereby contributing an adhesive and/or signalling function within this established stem cell niche [55].

The studies presented here also provide clear evidence that DSG2 is expressed uniformly by EPCs and by some mature ECs and that it regulates neoangiogenesis. First, we showed that DSG2 is expressed by an enriched population of non-adherent EPCs as well as freshly isolated CD34^+^CD45^dim^VEGFR2^+^ EPCs from blood. This finding is supported by the recent work of Patsch et al. [56] who converted human pluripotent stem cells into functional ECs and investigated their transcriptional gene signature. Our in silico analysis of this publically available gene expression array confirms that human ES cells express *DSG2* and reveals that this gene remains detectable, albeit to a lesser extent, in the transformed ECs (data not shown). Further interrogation of the endothelial lineage revealed that, while HUVEC are uniformly negative for DSG2, the human BM EC line (TrHBMEC) contains a distinct subpopulation that is DSG2 positive. This is consistent with our observations in normal human and cancerous tissues where, by immunohistochemistry and immunofluorescence, we detected DSG2 on some but not all blood vessels. Notably, we were able to detect DSG2 on both arterioles and venules and this is supported by our in silico analysis of the gene expression array conducted by Aranguren et al. [57] wherein we were able to detect *DSG2* in both arterial and venous ECs (data not shown). Interestingly, expression of *DSG2* within this cohort also varied across samples which supports our observation of differential expression of this cadherin by vascular ECs. Heterogeneity was also observed amongst mouse ECs when we performed an in silico analysis of a recent publication by Eugene Butcher’s laboratory, wherein a gene expression analysis was conducted on ECs isolated from mouse lymphoid organ HEVs and capillaries [41]. In support of our contention that certain ECs can express DSG2, Giusti et al. have also reported DSG2 expression by skin microvascular ECs [58]. It is tempting to speculate that DSG2 is expressed by newly formed vasculature, but the molecular basis underlying differential expression of DSG2 on ECs is yet to be elucidated and is part of ongoing studies within our laboratory.

A role for DSG2 in the vasculature is supported by our ‘loss-of-function’ *Dsg2*^lo/lo^ mice which show that *Dsg2* promotes BM-derived EPC colony formation, ex vivo vascular sprouting and formation of vessel-like structures in vitro and in vivo. These *Dsg2*^lo/lo^ mice were generated using a ‘knockout-first allele’ strategy which ablates gene function by insertion of a cassette into an intron of the intact gene and produces a knockout at the RNA processing level. This strain produced viable and fertile homozygous mice which displayed reduced, but not completely absent, levels of *Dsg2*; thus, our mice were termed *Dsg2* ‘hypomorphs’. The *Dsg2*^lo/lo^ mice described herein are comparable to those generated by Krusche et al. [43] who obtained largely fertile *Dsg2*-knockout mice but are distinct from the *Dsg2*-knockout mice published by Eshkind et al. [44], which were embryonic lethal at the time of blastocyst implantation. Importantly, the cardiomyopathy observed in the Krusche et al. [43] mice, characterised by fibrotic lesions in the heart, was also evident in our strain of *Dsg2*^lo/lo^ mice and is in keeping with the human clinical condition associated with *DSG2* ‘loss-of-function’, arrhythmogenic right ventricular cardiomyopathy/dysplasia (ARVC) [5, 6]. Fundamental differences between these three *Dsg2* ‘loss-of-function’ mice warrant further explanation; Eshkind et al. substituted exons 7 and 8 of the *Dsg2* gene with a cassette conferring *lacZ* expression and neomycin resistance in C57BL/6 and 129/Sv mouse strains, while Krusche et al. [59] utilised a targeting construct for inducible deletion of exons 4–6 in C57BL/6J mice. Our *Dsg2*^lo/lo^ mice differ again in that construct insertion is characterised by an in-frame intronic insertion between exons 1 and 2 on the C57Bl/6N background. It is possible that the positioning in intron 1 disrupts an enhancer region, resulting in ‘loss-of-function’, as has been documented for the epithelial cadherin (E-cadherin). To note, Eshkind et al. [44] demonstrated that the lethality of *Dsg2* depletion is influenced by genetic background of the mice, with 129/Sv mice displaying a higher lethality than the C57BL/6 strain.

Historically, studies of DSG2 function have focused on its role in desmosomes, with important clinical implications for understanding the pathogenesis of cardiac disease with increasing reports of dominantly inherited frameshift, splice site, nonsense and missense mutations of *DSG2* (mostly in the extracellular domain) in sudden death from ARVC as well as sudden unexplained death [60–62]. A recent study by Kant et al. showed that mice with cardiomyocyte-specific *Dsg2* ablation lack desmosome-like structures in the heart and that other desmosomal cadherins, e.g., Dsc2, are unable to fully compensate for the loss of Dsg2 [63]. However, with the identification of DSG2 on several normal and cancerous cell types that completely lack desmosomes [44, 64], there is increasing evidence for pleiotropic functions of this desmosomal cadherin. For example, Nava and colleagues demonstrated in intestinal epithelial cells that proteolytic cleavage of an intracellular fragment of DSG2 caused an increase in caspase 3 activation, cleavage of poly(ADP-ribose) polymerase (PARP) and cell apoptosis [65]. Brennan et al. also observed that overexpression of DSG2 in suprabasal keratinocytes induced hyperproliferation, resistance to anoikis and enhanced carcinogenesis [66]. Reports also document ‘non-junction-bound’ DSG2 on epithelial cells and cancer cells where it provides additional biological roles including regulation of apoptosis [65], proliferation [66, 67] and vasculogenic mimicry in melanoma [68]. Whether such effects are primary (a direct consequence of the change in DSG2 expression) or secondary (a response to changes in cell adhesion) is not certain. However, the ProteinPredict program of ExPASy indicates many potential protein kinase C (PKC)-target motifs exist in DSG2, including 13 in the cytoplasmic domain [69], and epidermal growth factor treatment of A431 epithelial cells induces tyrosine phosphorylation of DSG2 [70]. Moreover, the aforementioned Brennan et al. study of suprabasal expression of DSG2 in keratinocytes activated multiple pathways, including PI3K/Akt, MAPK/ERK, STAT3 and NFκB [66]. Thus, multiple lines of evidence suggest that DSG2 may contribute to intracellular signalling events in addition to its established role in cell adhesion.

In addition to the well-recognised link between *DSG2* mutation and cardiomyopathy in humans, a second clinical manifestation linked to *DSG2* loss-of-function has been identified in patients with systemic sclerosis (SSc, scleroderma). These patients exhibit insufficient angiogenesis in connective tissues, which is associated with a reduction in DSG2 expression in microvascular ECs [58]. This observation supports our contention that DSG2 regulates EC function and angiogenesis. The precise role for DSG2 in systemic sclerosis and angiogenesis is still unclear, with recent evidence suggesting impaired actin assembly and correlation with integrin β8 complex formation [71]. Interestingly, our observations contradict this study as we demonstrate that that loss of DSG2 by ECs induces stress fibre formation rather than impairing it. An important point of difference between these two studies is that we examined actin assembly in a confluent monolayer of ECs, while their coverage of ECs is clearly subconfluent, likely impacting on overall response and outcome. Herein we also show that loss of DSG2 by ECs interrupts VE-cadherin patterning within adherens junctions and culminates in a phenotype with heightened invasion through an extracellular matrix. Our adhesion assays suggest, for the first time, that DSG2 associates to form homotypic interactions between adjacent ECs, which form junctions designed to support vascular development. This is supported by documentation of DSG2–DSG2 interactions within the desmosome complex occurring via the ‘cell adhesion recognition’ (CAR) site. This conserved YAL sequence within the adhesive interface is located within the first of four extracellular repeat domains at the N-terminus of DSG2 and 10-mer peptides targeting this site have proven effective in their inhibition of epithelial cell interactions [72].

This study has revealed, for the first time, DSG2 expression in distinct progenitor cell subpopulations and shows that, independent from its canonical role as a component of desmosomes, this cadherin also plays a critical role in the vasculature. Because of this work, continued investigation of DSG2-mediated biology in EPCs and ECs, including the intercellular adhesive events, will undoubtedly provide further insight into the pathogenesis of vascular disease and the potential of manipulation of DSG2 for therapeutic use.

## Electronic supplementary material

Below is the link to the electronic supplementary material.

Supplementary material 1 (DOCX 17 kb)

Supplementary material 2 (PPT 332 kb)

Supplementary material 3 (DOCX 18 kb)

Supplementary material 4 (DOCX 14 kb)
