# Supplementary material for: A non-canonical role for desmoglein-2 in endothelial cells: implications for neoangiogenesis
Source: Angiogenesis. 2016 Jun 23;19(4):463–86. doi: 10.1007/s10456-016-9520-y (PMC5026727; doi:10.1007/s10456-016-9520-y)
Supplement: Supplementary file 1 — Supplementary material 1 (DOCX 17 kb) [file 10456_2016_9520_MOESM1_ESM.docx]

**ONLINE RESOURCE SUPPLEMENTARY FIGURE LEGENDS**

**Online resource Supplementary Figure 11: DSG2^+^ progenitor cells express CD117, CD133 and CD31 but not CD144.** MNC were isolated from UCB and stained for flow cytometric analysis. Cells were gated on the CD34^+^CD45^dim^DSG2^+^ population and then expression of CD117, CD133, CD31 or CD144 (solid line) determined relative to isotype controls (dotted line). Representative of n=6.

**Online resource Supplementary Figure 2: DSG2 is not expressed by mature leukocytes.** MNC were isolated from cord blood and stained for flow cytometric analysis. Cells were gated using lineage markers for the relevant leukocyte populations (CD3, CD16, CD14 or CD19). Within these populations, staining for DSG2 (solid line) was determined relative to an FMO control (dotted line). Representative of n=4.

**Online resource Supplementary Figure 3: DSG2 is not expressed by parental iPS cells.** Samples of two independent parental fibroblast cell lines (PDL, periodontal ligament and GF, gingival fibroblasts) as well as the iPS cells derived from them were stained for flow cytometric analysis. DSG2 expression (solid line) was distinguished from isotype control antibody (dotted line).
