# Supplementary material for: A non-canonical role for desmoglein-2 in endothelial cells: implications for neoangiogenesis
Source: Angiogenesis. 2016 Jun 23;19(4):463–86. doi: 10.1007/s10456-016-9520-y (PMC5026727; doi:10.1007/s10456-016-9520-y)
Supplement: Supplementary file 2 — Supplementary material 2 (PPT 332 kb) [file 10456_2016_9520_MOESM2_ESM.ppt]

## Slide 1
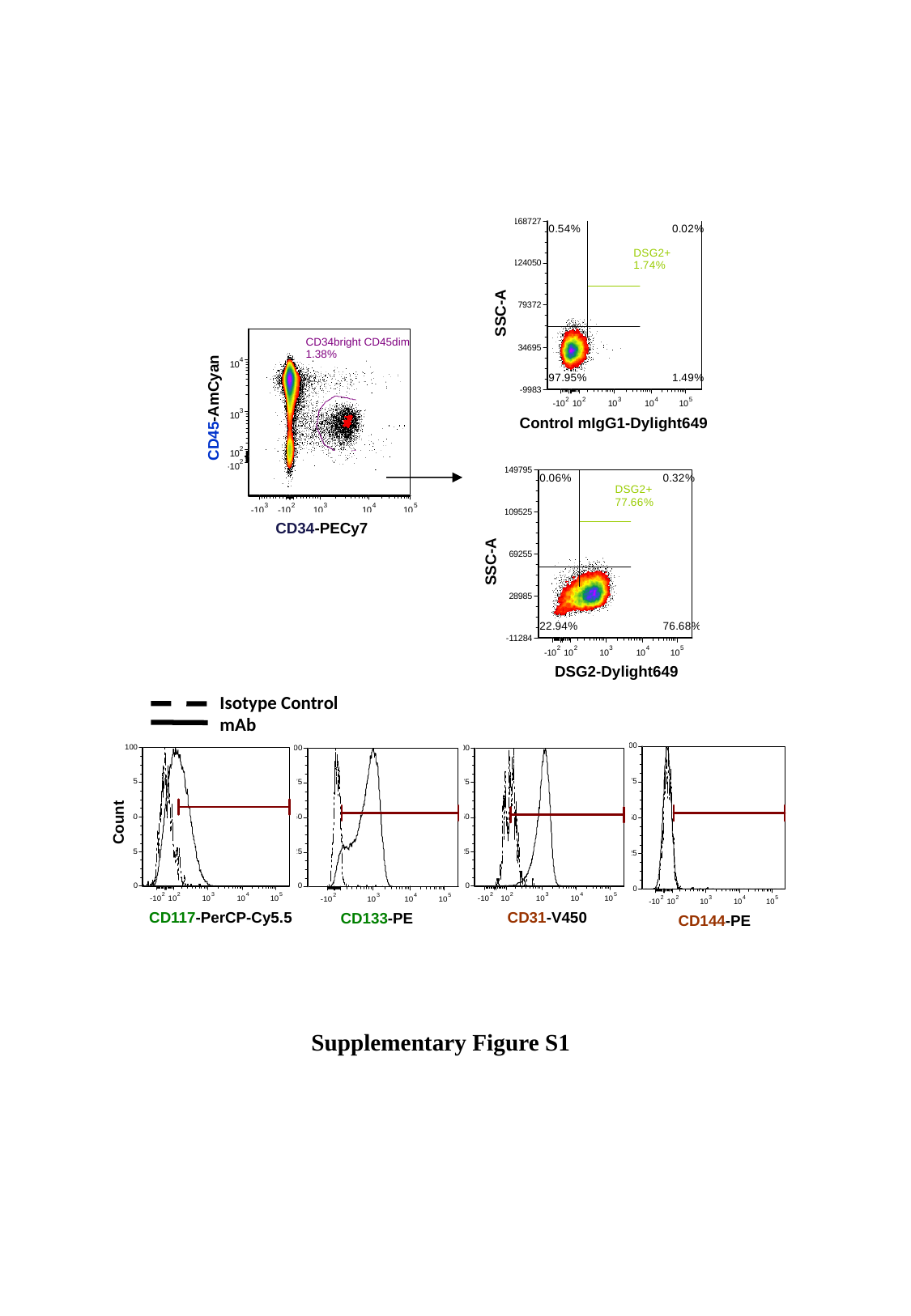

SSC-A
Control mIgG1-Dylight649
CD45-AmCyan
CD34-PECy7
SSC-A
DSG2-Dylight649
Isotype Control
mAb
Count
CD144-PE
Count
CD117-PerCP-Cy5.5
Count
CD133-PE
Count
CD31-V450
Supplementary Figure S1

## Slide 2
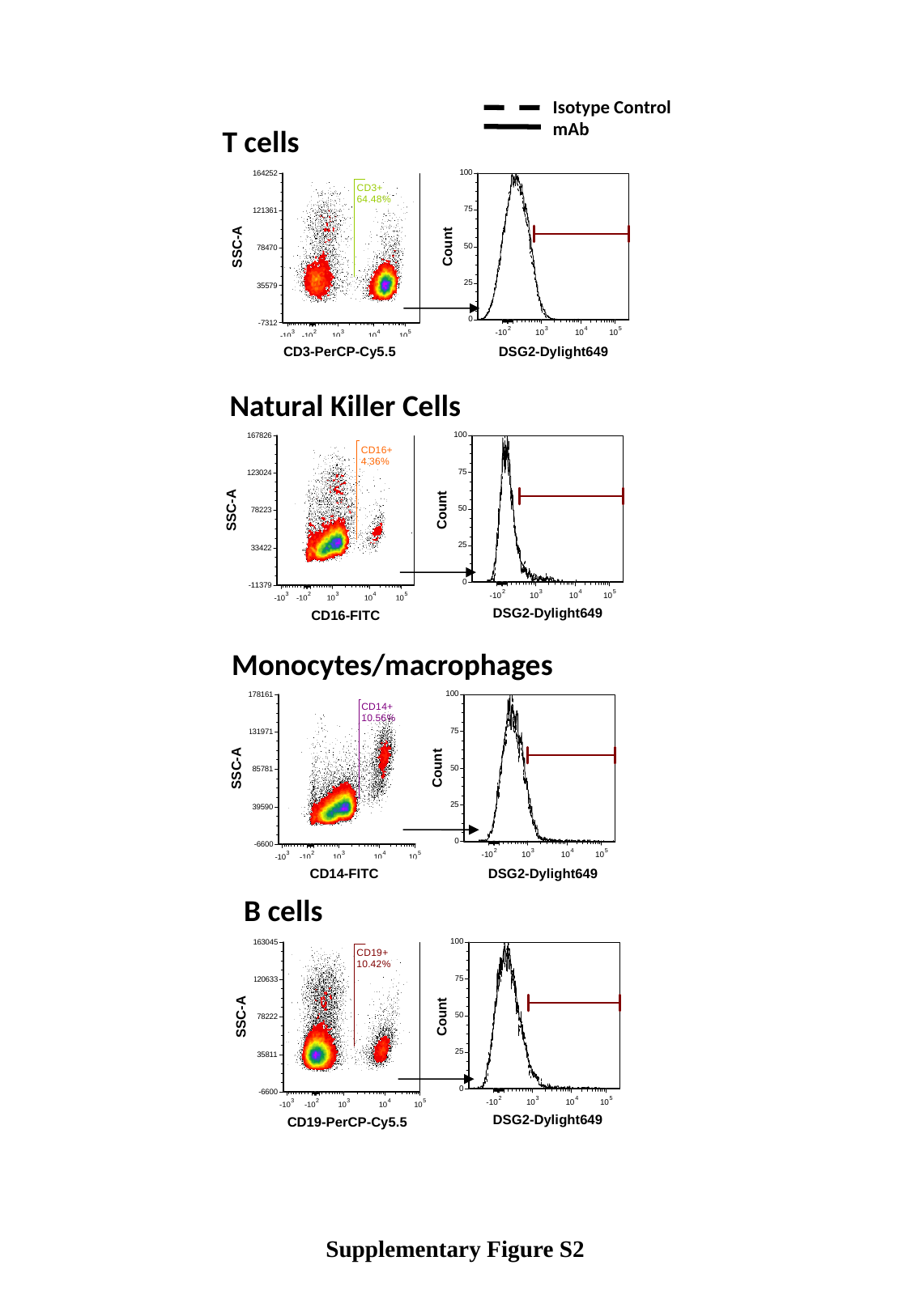

Isotype Control
mAb
T cells
SSC-A
Count
CD3-PerCP-Cy5.5
DSG2-Dylight649
Natural Killer Cells
SSC-A
Count
DSG2-Dylight649
CD16-FITC
Monocytes/macrophages
SSC-A
Count
CD14-FITC
DSG2-Dylight649
B cells
SSC-A
Count
DSG2-Dylight649
CD19-PerCP-Cy5.5
Supplementary Figure S2

## Slide 3
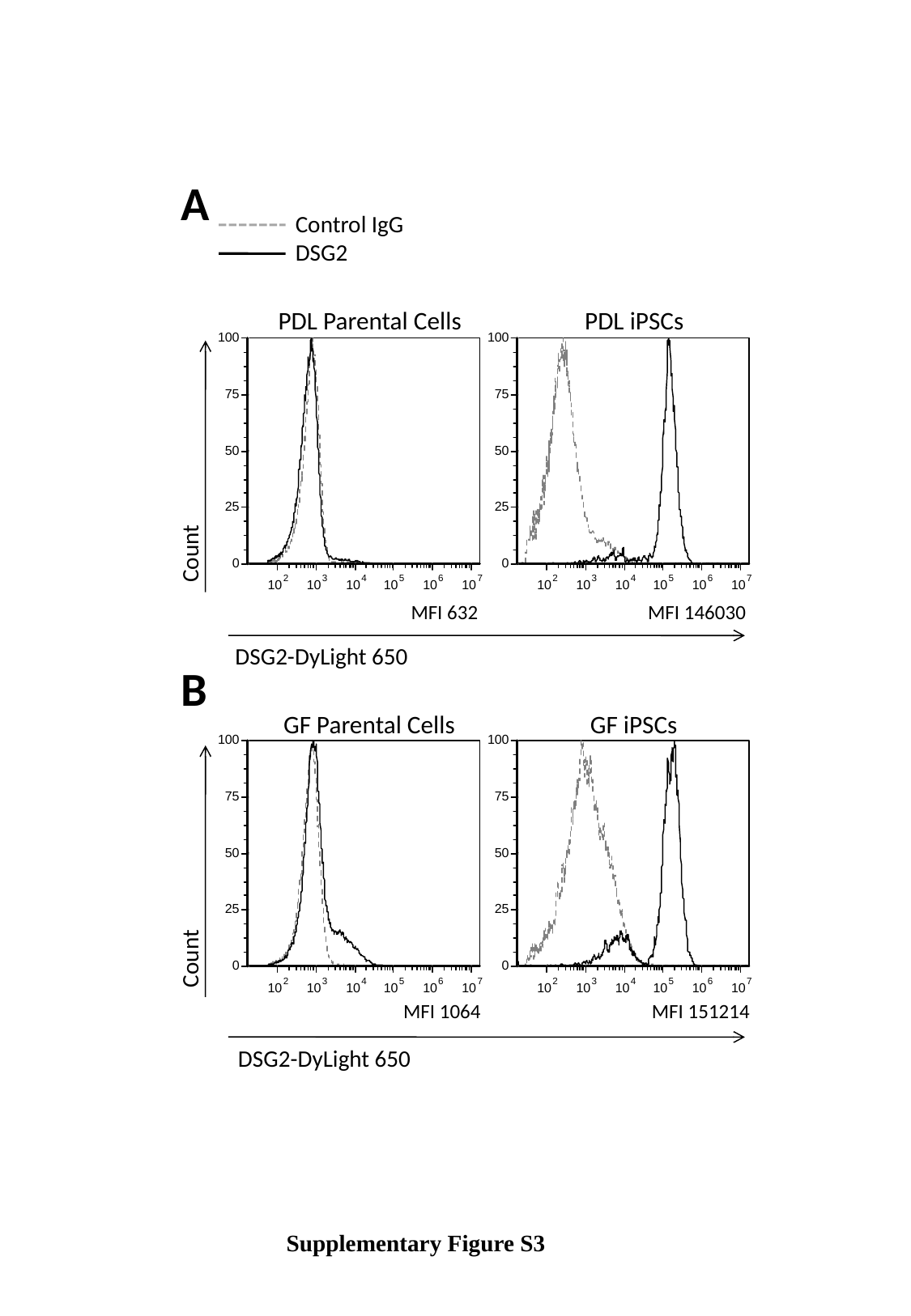

A
Control IgG
DSG2
PDL Parental Cells
PDL iPSCs
Count
MFI 632
MFI 146030
DSG2-DyLight 650
B
GF Parental Cells
GF iPSCs
Count
MFI 1064
MFI 151214
DSG2-DyLight 650
Supplementary Figure S3
