# Supplementary material for: A non-canonical role for desmoglein-2 in endothelial cells: implications for neoangiogenesis
Source: Angiogenesis. 2016 Jun 23;19(4):463–86. doi: 10.1007/s10456-016-9520-y (PMC5026727; doi:10.1007/s10456-016-9520-y)
Supplement: Supplementary file 3 — Supplementary material 3 (DOCX 18 kb) [file 10456_2016_9520_MOESM3_ESM.docx]

**Online resource Supp. Table 11: antibodies used in flow cytometry**

| **Specificity** | **Conjugate** | **Clone** | **Supplier** | **Used in** |
| --- | --- | --- | --- | --- |
| *Primary antibodies* | | | | |
| CD10 | PECy5 | HI10a | BD | Figure 3 |
| CD11b | PE | D12 | BD | Figure 3 |
| CD13 | PECy7 | L138 | BD | Figure 3 |
| CD14 | APC | M5E2 | BD | Figure 3 |
| CD16 | FITC | 3G8 | Beckman Coulter | Figure 3 |
| CD19 | PECy7 | HIB19 | BD | Figure 3 |
| CD20 | APC | L27 | BD | Figure 3 |
| CD22 | PE | 4KB128 | Dako | Figure 3 |
| CD33 | PECy5 | WM53 | BD | Figure 3 |
| CD34 | FITC | 8G12 | BD | Figure 3 |
| CD34 | PerCPCy5.5 | 8G12 | BD | Figure 4 |
| CD34 | PECy7 | 8G12 | BD | Figure 2 |
| CD38 | FITC | HB7 | BD | Figure 3 |
| CD45 | APC-H7 | 2D1 | BD | Figure 3 |
| CD45 | AmCyan | 2D1 | BD | Figure 2 |
| CD45 | FITC | 2D1 | BD | Figure 4 |
| CD71 | FITC | LO1.1 | BD | Figure 3 |
| CD90 | PE | 5E10 | BD | Figure 3 |
| CD117 | APC | YB5.B8 | BD | Figure 3 |
| CD235a | PE | GA-R2 | BD | Figure 3 |
| DSG1 | unconjugated | 27B2 | Life Technologies | Figure 2 |
| DSG2 | unconjugated | 6D8 | Life Technologies | Figures 1-5,9 |
| DSG3 | unconjugated | 5G11 | Life Technologies | Figure 2 |
| DSC2/3 | unconjugated | 7G6 | Life Technologies | Figure 2 |
|  |  |  |  |  |
| *Secondary antibodies* | | | | |
| mouse IgG | PECy7 | polyclonal | Santa Cruz | Figure 1, 5 |
| mouse IgG | Brilliant Violet 421 | polyclonal | BioLegend | Figures 2-3 |
| mouse IgG | PE | polyclonal | Southern Biotech | Figure 4 |
