# Supplementary material for: A non-canonical role for desmoglein-2 in endothelial cells: implications for neoangiogenesis
Source: Angiogenesis. 2016 Jun 23;19(4):463–86. doi: 10.1007/s10456-016-9520-y (PMC5026727; doi:10.1007/s10456-016-9520-y)
Supplement: Supplementary file 4 — Supplementary material 4 (DOCX 14 kb) [file 10456_2016_9520_MOESM4_ESM.docx]

**Online resources Supp. Table 2: Expression of DSG2 by human normal and cancerous tissue.**

| **Normal tissues with detectable vessels** | | **Tumor tissues with detectable vessels** | |
| --- | --- | --- | --- |
| *DSG2+ vessels*  *detected* | *All vessels*  *DSG2-* | *DSG2+ vessels*  *detected* | *All vessels DSG2-* |
|  |  |  |  |
| Adrenal  Uterus  Cervix  Esophagus  Small intestine  Thymus  Placenta  Greater omentum | Lung  Colon  Salivary gland  Cerebellum  Spleen  Tonsil  Pericardium  Peripheral nerve | Lung carcinoma  Breast carcinoma  Colon carcinoma  Liver carcinoma  Kidney carcinoma  Sarcoma  Neurofibroma  Neuroblastoma | Esophageal carcinoma  Intestinal adenocarcinoma  Ovarian carcinoma  Glioma  Meningioma  Lymphoma  Bladder carcinoma |
